# *Oxalis corniculata* L. Ethanol Extract Promotes Fracture Healing: Integrated Omics and Experimental Validation

**DOI:** 10.1002/fsn3.71896

**Published:** 2026-05-19

**Authors:** Jian Zhang, Xiaorong Zhao, Xiaoyan Long, Yu Zhang, Xuegang Luo, Wanyan Shen

**Affiliations:** ^1^ College of Life Sciences and Agri‐Forestry Southwest University of Science and Technology Mianyang China; ^2^ GuiZhou Institute of Subtropical Crops Guizhou Academy of Agricultural Sciences Guiyang China; ^3^ Engineering Research Center of Biomass Materials, Ministry of Education Southwest University of Science and Technology Mianyang China; ^4^ Wangcang County Hospital of Traditional Chinese Medicine Guangyuan China; ^5^ Research and Development Department Guizhou Weikang Zifan Pharmaceutical co., Ltd. Guiyang China

**Keywords:** experimental validation, fracture, network pharmacology, *Oxalis corniculata* L.

## Abstract

*Oxalis corniculata* L. is a well‐known herb valued for its dual edible and medicinal properties, with its acidic compounds endowing food with a distinct flavor and enhancing its value. This study identified the active compounds in its ethanol extract (OCEE) and clarified the molecular mechanisms underlying its fracture‐healing effects. UHPLC‐Q‐Exactive‐MS/MS was used for comprehensive chemical profiling of OCEE, and network pharmacology was applied to explore therapeutic targets and signaling pathways related to fracture treatment. Molecular docking (MD), molecular dynamics simulations (MDS), and in vitro cell assays further validated the core mechanisms. A total of 549 compounds were identified in OCEE, with naringenin‐7‐O‐glucuronide, chrysophanol‐8‐O‐glucoside, and 2‐oxo‐3‐phenylpropanoic acid as key potential active components. These compounds targeted GAPDH, TNF, TP53, and other core proteins, which are involved in nitrogen metabolism, IL‐17 signaling, and arachidonic acid metabolism pathways critical for fracture healing. MD and MDS confirmed strong binding affinities between key compounds and core targets, and qPCR results revealed that OCEE significantly upregulated the expression of TNFα, MMP9, and ESR1 while downregulating TP53 expression in vitro. These findings clarify the material basis and molecular pathways of OCEE in promoting fracture healing and provide experimental evidence for its application as a functional food and natural agent for bone health and fracture recovery.

## Graphical abstract

*Oxalis corniculata* L. ethanol extract (OCEE) showed efficacy in promoting fracture healing. A total of 549 compounds were identified by UHPLC‐Q‐Exactive‐MS/MS, with three key components recognized. OCEE may accelerate fracture healing by regulating TNFα, MMP9, ESR1, TP53, and related signaling pathways.

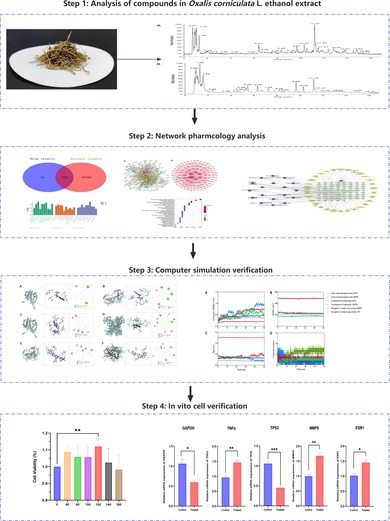

## 1 Introduction

*Oxalis corniculata* L., a plant renowned for its dual role as both a food and medicinal resource, exhibits unique edible and medicinal properties. The acidic compounds in this plant contribute unique flavor profiles to food products. In Guizhou Province, China, it is locally referred to as “Suan Mi Mi.” Traditionally, *Oxalis corniculata* L. has served as a therapeutic agent in Chinese medicine and appears officially in the “Quality Standards of Traditional Chinese and Ethnic Medicinal Materials in Guizhou Province” (Guizhou Medical Products Administration 2003). Both fresh and dried forms of the plant are known for their medicinal properties, with its sour taste linked to effects such as heat clearance, detoxification, anti‐inflammatory action, and pain relief (Zhang et al. 2024). *Oxalis corniculata* L. contains various bioactive constituents, encompassing flavonoids, phenolic acids, alkaloids, volatile oils, terpenes, and saponins, which exhibit anti‐inflammatory, edema‐reducing, and injury‐healing properties (Sreejith et al. 2014; Kiran et al. 2024).

Fractures are characterized by the disruption of bone integrity, resulting in broken bones, deformities, abnormal movement, crepitus, and common symptoms including pain, tenderness, localized swelling, and bruising (Einhorn and Gerstenfeld 2015; Ferrari et al. 2026; Mårild et al. 2024). In severe cases, fractures may lead to shock or pose life‐threatening risks. Within the bone marrow, mesenchymal stem cells can differentiate into osteoblasts, adipocytes, or chondrocytes, depending on the local microenvironment (Huang et al. 2024). Osteoblasts are pivotal in the fracture healing process, primarily responsible for bone formation (Ponzetti and Rucci 2021). Upon fracture, osteoblasts become activated and migrate toward the injury site, where they release bone matrix proteins such as collagen, initiating the formation of new bone tissue (Capulli et al. 2014). Furthermore, osteoblasts regulate the deposition of minerals like calcium and phosphorus, contributing to the mineralization and hardening of newly formed bone (Bottini et al. 2018). Herbal medicines exert a key role in the management of chronic degenerative diseases due to their unique holistic concept and multi‐dimensional therapeutic strategies. Products such as Gukang capsules and Zhongtongshu sprays, which contain *Oxalis corniculata* L., have been developed and shown to effectively treat osteoporosis, fractures, and injuries (Zhu et al. 2022).

Network pharmacology (NP), an interdisciplinary domain combining systems biology, bioinformatics, and network analysis, constructs the interaction network between drugs and the biological system, elucidating the drug's mechanisms of action (Ru et al. 2014). This approach offers a novel method and perspective for researching traditional Chinese medicine, facilitating enhanced comprehension of its mechanisms and assisting in the identification of bioactive compounds in Chinese medicinal herbs (Zhang et al. 2025). *Oxalis corniculata* L. can promote fracture healing in rabbits, potentially through mechanisms such as upregulation of ALP activity, reduction of blood viscosity, and improvement in hemorheological properties (Tang et al. 2022). Nevertheless, the precise molecular mechanisms underlying its fracture‐healing effects remain poorly understood and have not been systematically documented. Moreover, existing research on herbal extracts for fracture healing often lacks a comprehensive approach that combines high‐throughput chemical profiling, in silico target prediction, and experimental validation. Few studies have clarified the material basis of *Oxalis corniculata* L. ethanol extract (OCEE) in promoting fracture healing. This gap hampers the translation of this plant into functional food resources for bone health. Therefore, given the limited research on the effective substances and underlying mechanisms of *Oxalis corniculata* L. in fracture healing, this study employed UHPLC‐Q‐Exactive‐MS/MS to identify the chemical components of OCEE. Furthermore, network pharmacology combined with experimental validation was used to explore its potential mechanisms in promoting fracture healing, which may provide a scientific basis for the development of related functional foods and pharmaceuticals.

## 2 Materials and Methods

### 2.1 Reagents

The *Oxalis corniculata* L. powder was sourced from Guizhou Weikang Zifan Pharmaceutical Co. Ltd. Analytical‐grade chemicals, including methanol, acetonitrile, formic acid, and isopropanol (acquired from Anpu), were used in the experiment, along with 95% ethanol (analytical grade, Shanghai Shenggong). Ultrapure water was employed as the water source.

### 2.2 Sample Analysis

#### 2.2.1 Sample Preparation

Forty‐five percent ethanol was selected as the extraction solvent based on the traditional folk usage of fracture‐healing herbs and its efficiency in enriching bioactive flavonoids and phenolic compounds. For extract preparation, 1 g of *Oxalis corniculata* L. powder was extracted with 45% ethanol at a solid–liquid ratio of 1:10 g/mL (initial 2 mL for 30 min, followed by 8 mL for 6 h) under natural pH without adjustment. Subsequently, 1 mL supernatant was combined with 2 mL methanol‐acetonitrile solution (1:1, v/v). The mixture was vortexed for 60 s, then subjected to low‐temperature ultrasonic processing for 30 min. Centrifugation was performed at 12,000 rpm and 4°C for 10 min, after which the sample remained at −20°C for 1 h to promote protein precipitation. Subsequently, another centrifugation step was conducted at 12,000 rpm and 4°C for 10 min. The obtained supernatant underwent freeze‐drying and was subsequently resuspended in 100 μL of 50% acetonitrile, followed by vortexing and centrifugation at 12,000 rpm and 4°C for 10 min. The final supernatant was employed for analysis.

#### 2.2.2 LC–MS Conditions

During analysis, samples were maintained at 4°C in an auto‐sampler, employing a 2 μL injection volume. A Waters HSS T3 column (100 × 2.1 mm, 1.8 μm) was used, held at 40°C. The mobile phases encompassed 0.1% formic acid in water (designated as A) and 0.1% formic acid in acetonitrile (designated as B). The flow rate was established at 0.3 mL/min. The elution gradient proceeded as follows: from 0.0 to 1.0 min, 0% B was held constant; from 1.0 to 12.0 min, B increased from 0% to 95%; from 12.0 to 13.0 min, B was held at 95%; from 13.0 to 13.1 min, B declined from 95% to 0%; and from 13.1 to 17.0 min, 0% B was held constant.

For mass spectrometry analysis, a Thermo Scientific Q Exactive HFX high‐resolution mass spectrometer conducted primary and secondary scans. The ESI parameters were configured as follows: sheath gas flow rate at 40 arb, auxiliary gas flow rate at 10 arb, ion spray voltage at 3000 V/−2800 V, temperature at 350°C, and ion transfer tube temperature at 320°C. The scanning mode utilized Full‐ms‐ddMS2, enabling detection of both positive and negative ions. The primary scan range covered 70–1050 Da, with resolution configurations of 70,000 for primary scans and 17,500 for secondary scans. Compound identification was performed using Sanshu Biotech's proprietary TCM database with secondary MS/MS fragmentation pattern matching. All metabolites were assigned as MSI Level 2 by matching against the database.

### 2.3 NP Study

#### 2.3.1 Target Compound Acquisition

The compounds present in OCEE and their structural details were procured from the NCBI PubChem database (https://pubchem.ncbi.nlm.nih.gov/). The compounds' isomeric SMILES structures were then entered into the Swiss Target Prediction database (http://www.swisstargetprediction.ch/) for potential target identification. Subsequently, these targets underwent mapping to gene names via the UniProt database (https://www.uniprot.org/), establishing a comprehensive list of OCEE‐related targets.

#### 2.3.2 Fracture Target Acquisition

To identify genes associated with fractures, the keyword “Fracture” was used to extract relevant genes from three disease gene databases: GeneCards (https://www.genecards.org/), CTD (https://ctdbase.org/), and OMIM (https://omim.org/). The collected data underwent curation, and genes common across databases were designated as fracture‐related disease targets.

#### 2.3.3 Prediction of OCEE on Fracture Targets

Utilizing the online tool Venny (https://bioinfogp.cnb.csic.es/tools/venny/), the OCEE‐related targets were compared with the fracture‐related disease targets. The overlapping targets between these two groups were considered as the effective therapeutic targets of OCEE in fracture treatment.

#### 2.3.4 Elaboration of the Protein–Protein Interaction (PPI) Network

Utilizing the STRING data analysis platform (https://string‐db.org/), the intersecting genes were systematically analyzed to construct a comprehensive PPI network, resulting in the creation of the “OCEE‐Fracture Targets” network. This network underwent additional analysis through the Cytohubba plugin within Cytoscape software for selecting core targets according to their interaction scores.

#### 2.3.5 GO and KEGG Pathway Enrichment Analyses

Gene Ontology (GO) and Kyoto Encyclopedia of Genes and Genomes (KEGG) enrichment analyses were executed on the core targets using R software. These analyses sought to clarify OCEE's potential biological functions and essential signaling pathways in fracture healing. A strict *q* value threshold of < 0.05 was applied, and the findings were organized according to decreasing *p* value to highlight the most significant enrichments.

### 2.4 Computer Simulation Verification

#### 2.4.1 Molecular Docking (MD)

Core compounds from OCEE were imported into Chem3D software for the construction of their chemical structures, followed by energy minimization, and saved in PDB format. Relevant human targets were retrieved from the PDB database, including GAPDH (PDB ID: 6M61), TNF (PDB ID: 1TNF), TP53 (PDB ID: 2BIM), MMP9 (PDB ID: 1GKC), and ESR1 (PDB ID: 1A52). Water molecules and non‐target ligands were removed before analysis. For blind docking, the grid box was set to 120 × 120 × 120 Å to cover the entire protein surface. Molecular docking and visualization were carried out using all‐atom docking methods in PyMOL and MOE software. The MOE scoring function was applied to evaluate binding affinities.

#### 2.4.2 Molecular Dynamics Simulation (MDS) Experiment

Gromacs v2022.03 was utilized to perform 50 ns MDS on protein‐small molecule complexes procured from MD, employing the AMBER99SB‐ILDN force field (Lindorff‐Larsen et al. 2010). Key structural and energetic parameters, encompassing root mean square deviation (RMSD), solvent‐accessible surface area (SASA), radius of gyration (Rg), and hydrogen bonds (H‐bonds), underwent analysis from the MDS trajectories. The system's Gibbs free energy underwent calculation utilizing the “g_sham” and “xpm2txt.py” scripts in Gromacs v2022.03, derived from RMSD and Rg data. Furthermore, binding free energy calculations for protein‐small molecule complexes underwent execution using the “MMPBSA.py v.16.0” script (Genheden and Ryde 2015).

### 2.5 Cell Experiments Verification

#### 2.5.1 Cell Culture

Human osteoblasts (hFOB1.19, Shanghai Cell Bank of the Chinese Academy of Sciences) were thawed, with subculturing initiated once cells achieved 80%–95% confluence. Cells were resuspended using preheated 0.25% Trypsin–EDTA medium (Thermo Fisher, USA) at 37°C.

#### 2.5.2 Cell Proliferation Assay

hFOB1.19 cells during the logarithmic growth phase were collected and placed into 96‐well plates at a density of 3 × 10^3^ cells per well. OCEE treatment was applied at concentrations of 0, 60, 80, 100, 120, 140, and 160 μg/mL. Control group received blank medium, with six replicates for each treatment group. After a 48‐h culture period, each well received 10 μL CCK‐8 reagent and underwent 1.5‐h incubation. A microplate reader measured absorbance at 450 nm, enabling calculation of cellular proliferation rates for individual experimental groups.

#### 2.5.3 qPCR Analysis of Key Gene Expression Levels

Total RNA extraction from cells was executed utilizing TRIzol Reagent (Life Technologies, USA), followed by cDNA synthesis. qPCR assays were performed on the CFX384 PCR instrument (Bio‐Rad, USA). The PCR amplification program was as follows: an initial step at 95°C for 30 s, then 40 cycles comprising 95°C for 5 s and 60°C for 30 s. β‐actin served as the internal reference gene, with relative mRNA expression levels determined through the 2^−ΔΔCT^ methodology. qPCR primers were developed utilizing Primer Premier 6.0 and Beacon Designer 7.8 software, followed by synthesis through Sangon Biotech Shanghai Co. Ltd. Their sequences are provided in Table 1.

**TABLE 1:** Primers for qPCR.

Gene	Forward primer	Reverse primer
β‐actin	GATGACCCAGATCATGTTTGAGAC	GGAGTCCATCACGATGCCAGT
GAPDH	CCATGACAACTTTGGTATCGTGGAA	GGCCATCACGCCACAGTTTC
TNFα	CCATGTTGTAGCAAACCCTCAAGCT	CCTTGAAGAGGACCTGGGAGTAGAT
TP53	CGTGAGCGCTTCGAGATGTT	ACTTCAGGTGGCTGGAGTGA
MMP9	GCCACTACTGTGCCTTTGAGTC	CCCTCAGAGAATCGCCAGTACT
ESR1	GCTTACTGACCAACCTGGCAGA	GGATCTCTAGCCAGGCACATTC

### 2.6 Statistical Analysis

Data analysis was performed using GraphPad Prism 10.1.2. The Shapiro–Wilk test was applied to assess normality prior to analysis. For the cell proliferation assay, as some groups did not meet the normality assumption, the Kruskal–Wallis test followed by Dunn's post hoc test was used for multi‐group comparisons. For qPCR analysis, the unpaired Student's *t*‐test was used for genes where both datasets passed the normality test, while the Mann–Whitney *U* test was applied for GAPDH where the control group violated the normality assumption. All data are presented as mean ± SD, and *p* < 0.05 was considered statistically significant.

## 3 Results

### 3.1 Compounds Identification in OCEE

**FIGURE 1:**
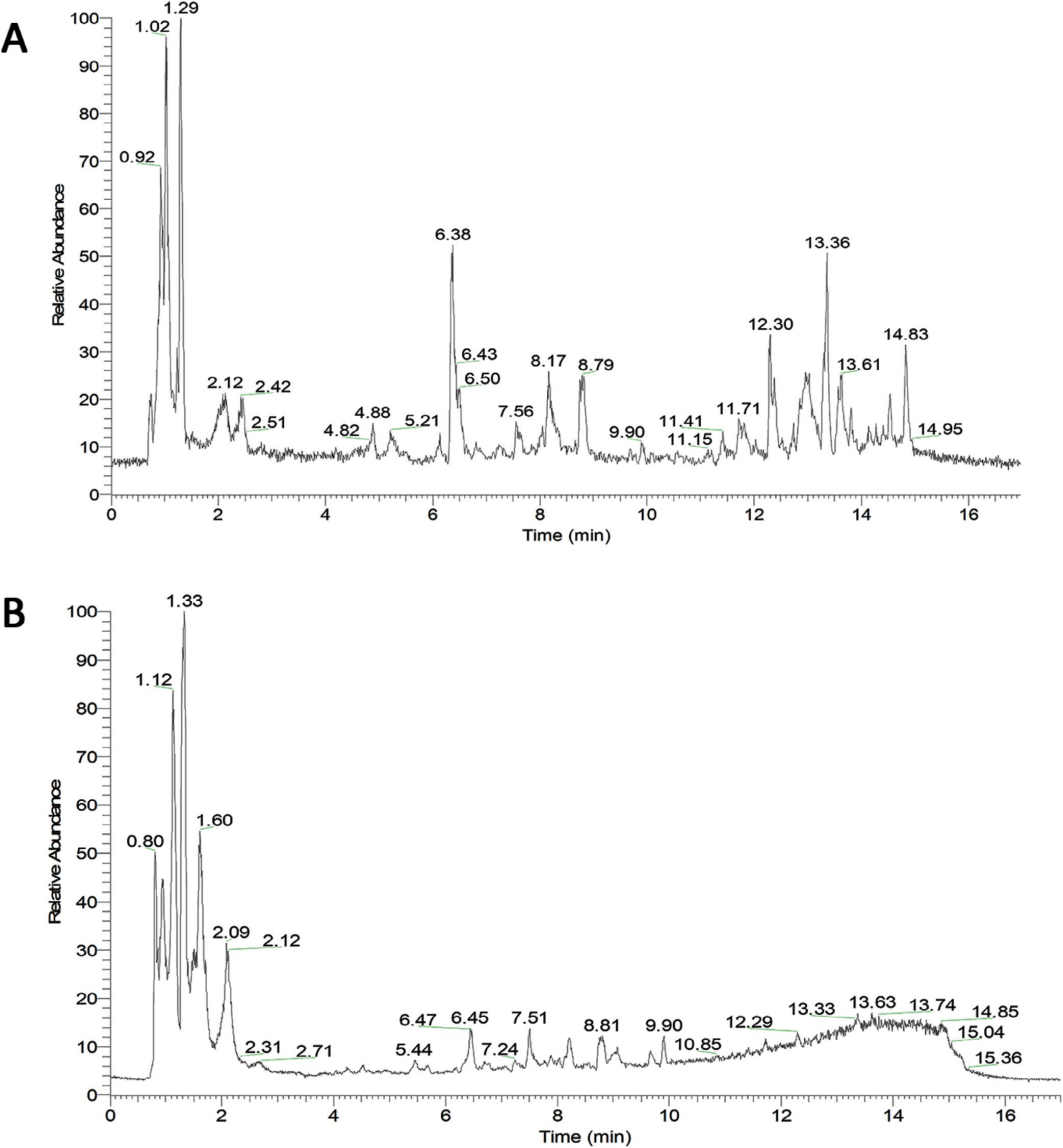
TIC diagrams of positive ion (A) and negative ion (B) modes of OCEE.

Using Sanshu Biotech's proprietary database focusing on TCM, a total of 549 chemical compounds were identified. Detailed information is provided in Table S1. In positive ion mode, 323 compounds were detected (Figure 1A), while 226 compounds were detected in negative ion mode (Figure 1B). These compounds were split into distinct groups, comprising 159 phenylpropanoids and polyketides, 94 lipids and lipid‐like molecules, 64 organic oxygen compounds, 59 organoheterocyclic compounds, 46 benzenoids, 38 organic acids and derivatives, 13 nucleosides, nucleotides, and analogues, 4 alkaloids and derivatives, 4 lignans, neolignans, and related compounds, 2 hydrocarbon derivatives, 2 organic nitrogen compounds, and 64 other compounds.

Based on the relative peak areas, the top 20 compounds with the highest relative contents in OCEE were selected for further experiments (Table 2). Through the Swiss Target Prediction database, 136 drug targets linked to OCEE were systematically determined.

**TABLE 2:** Details regarding the top 20 compounds in terms of relative contents within OCEE.

No.	Compound	Formula	Rt (min)	m/z	Adducts	CAS
1	10‐O‐Coumaroyl‐10‐O‐deacetylasperuloside	C_25_H_26_O_12_	8.04	563.1403	M + FA‐H	903519‐82‐4
2	3′‐hydroxyPuerarin	C_21_H_20_O_10_	8.75	433.1122	M + H, M + Na, 2 M + Na, M + H‐H_2_O, M + H‐2H_2_O	117060‐54‐5
3	Naringenin‐7‐O‐beta‐D‐glucuronide	C_21_H_20_O_11_	8.23	449.1070	M + H‐2H_2_O, M + H, M + Na, M + H‐H_2_O	529‐55‐5
4	Isovitexin	C_21_H_20_O_10_	8.75	431.0978	M‐H	38953‐85‐4
5	D‐proline	C_5_H_9_NO_2_	1.03	116.0708	M + H, M + K, M + ACN + H, 2 M + H	344‐25‐2
6	Isoorientin	C_21_H_20_O_11_	8.23	447.0929	M‐H_2_O‐H, 2M‐H, M‐H	4261‐42‐1
7	4″‐methyloxy‐Genistin	C_22_H_22_O_10_	8.82	445.1135	M‐H, 2M‐H	950910‐16‐4
8	Isoschaftoside	C_26_H_28_O_14_	8.54	563.1403	M‐H	52012‐29‐0
9	Emodin‐8‐O‐beta‐gentiobioside	C_27_H_30_O_15_	7.60	593.1510	M‐H	66466‐22‐6
10	Betaine	C_5_H_11_NO_2_	0.94	118.0864	M + H	107‐43‐7
11	Kaempferol 3‐glucoside 7‐rhamnoside	C_27_H_30_O_15_	7.60	595.1649	M + H, M + Na, M + 2Na‐H, M + H‐H_2_O	66465‐24‐5
12	Ruberythric acid	C_25_H_26_O_13_	7.72	579.1352	M + FA‐H	152‐84‐1
13	N‐Acetyl‐Neuraminic Acid	C_11_H_19_NO_9_	1.67	290.0877	M‐H_2_O‐H, M‐H	131‐48‐6
14	Panasenoside	C_27_H_30_O_16_	7.24	611.1599	M + H, M + Na, M + H‐H_2_O	31512‐06‐8
15	2‐Oxo‐3‐phenylpropanoic acid	C_9_H_8_O_3_	6.51	147.0439	M + H‐H_2_O	156‐06‐9
16	Chrysophanol 8‐O‐glucoside	C_21_H_20_O_9_	8.85	461.1085	M + FA‐H	13241‐28‐6
17	N‐a‐Acetyl‐L‐arginine	C_8_H_16_N_4_O_3_	1.30	280.1385	M + H‐2H_2_O, M + H, M + ACN + Na	155‐84‐0
18	D‐altrofurano‐heptulose‐3	C_7_H_14_O_7_	0.95	245.0428	M + Cl	25545‐06‐6
19	Trigonelline	C_7_H_7_NO_2_	1.01	138.0549	M + ?	535‐83‐1
20	Rubrofusarin‐6‐O‐beta‐D‐gentiobioside	C_27_H_32_O_15_	8.71	577.1559	M‐H_2_O‐H	24577‐90‐0

### 3.2 NP Results

#### 3.2.1 Intersectional Analysis Between OCEE Targets and Fracture‐Associated Genes

**FIGURE 2:**
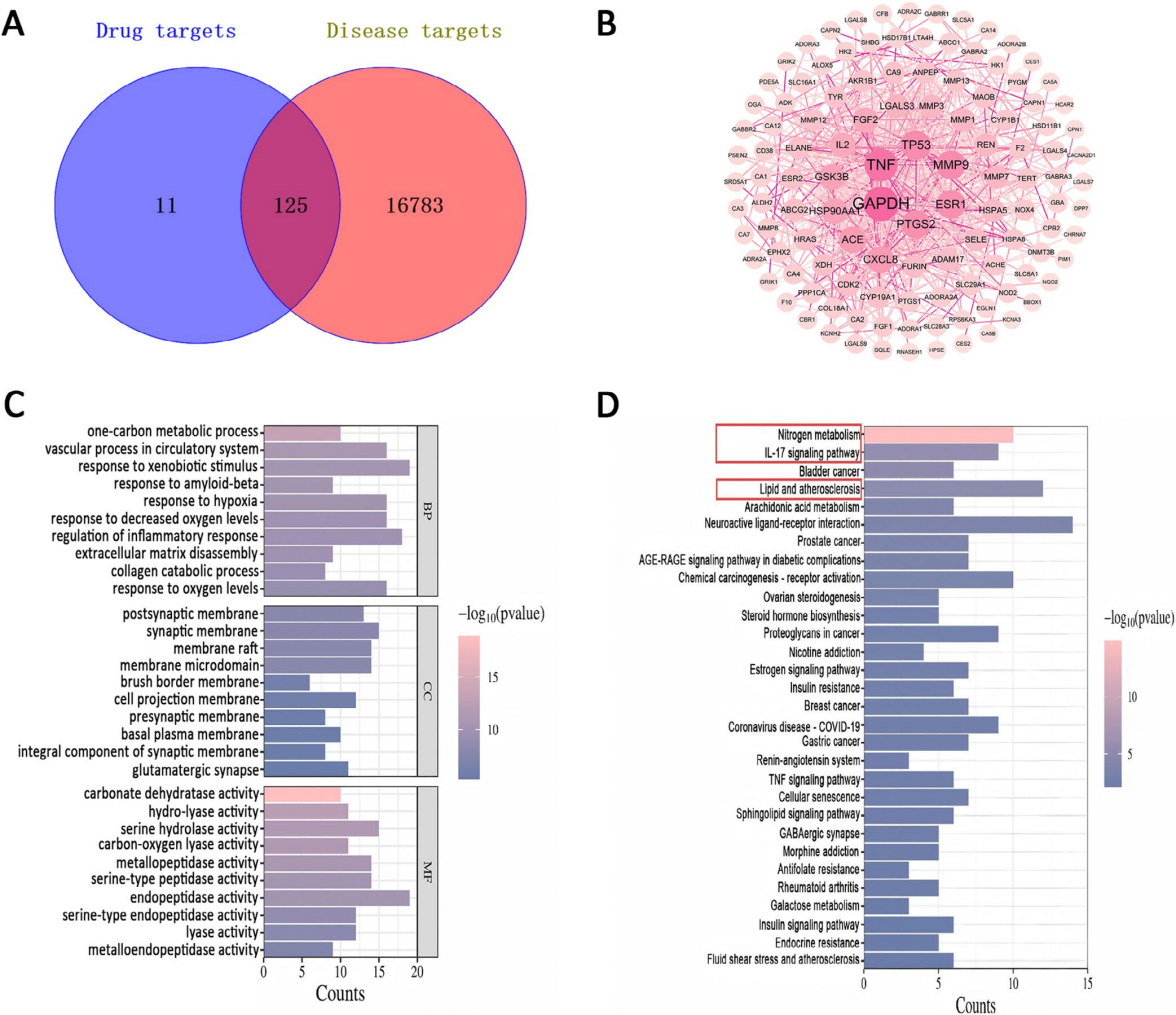
Bioinformatics analysis of key targets for OCEE in fracture healing. (A) Venn diagram of common targets between OCEE and fracture. (B) PPI network of core targets. (C) Histogram of GO functional enrichment analysis. (D) KEGG pathway enrichment analysis of core targets.

Bioinformatics analysis of the key targets of OCEE in fracture healing was shown in Figure 2. Through a deduplication process utilizing the GeneCards, CTD, and OMIM databases, 16,908 genes related to fractures were identified. Intersectional analysis with OCEE target genes revealed 125 potential targets involved in fracture treatment, as shown in Figure 2A.

#### 3.2.2 PPI Network for OCEE in Fracture Treatment

Using the STRING database, a detailed analysis of interactions among OCEE targets for fracture treatment was conducted (Figure 2B). Core targets were determined utilizing the Cytohubba plugin within Cytoscape software, employing criteria including Degree ≥ 10.78991597, Betweenness Centrality ≥ 0.013547879, and Closeness Centrality ≥ 0.402693092, yielding 19 core targets, as presented in Table 3.

**TABLE 3:** Topological parameter analysis of core targets.

Target	Degree	Betweenness centrality	Closeness centrality
GAPDH	62	0.244973	0.637838
TNF	55	0.142843	0.592965
TP53	41	0.060580	0.538813
MMP9	41	0.061498	0.538813
ESR1	39	0.064749	0.531532
PTGS2	39	0.045453	0.531532
CXCL8	33	0.015478	0.508621
ACE	32	0.068223	0.515284
GSK3B	29	0.042222	0.508621
HSP90AA1	29	0.050304	0.502128
LGALS3	22	0.019665	0.468254
ABCG2	16	0.058105	0.472000
CYP19A1	16	0.031039	0.459144
ESR2	15	0.019913	0.470120
AKR1B1	14	0.016729	0.468254
ANPEP	14	0.019041	0.445283
CA9	14	0.033367	0.459144
MAOB	13	0.055701	0.466403
ACHE	11	0.025273	0.452107

#### 3.2.3 GO and KEGG Analyses

Utilizing the clusterProfiler package in R software, comprehensive GO functional enrichment analysis was executed on the 125 intersecting targets of OCEE in fracture treatment. The analysis identified 1358 GO terms (*q* value < 0.05) spanning Biological Processes (BP), Cellular Components (CC), and Molecular Functions (MF). Figure 2C displays the top 10 GO terms. Of these, 1160 BP terms were identified, primarily related to one‐carbon metabolic processes, circulatory system vascular functions, responses to exogenous stimuli, β‐amyloid responses, and hypoxia responses. The CC analysis identified 61 terms, focusing mainly on post‐synaptic membranes, synaptic membranes, membrane rafts, membrane microdomains, and brush border membranes. Additionally, 137 MF terms were identified, predominantly linked to carbonic anhydrase activity, hydrolase activity, serine hydrolase activity, carboxy‐lyase activity, and metalloproteinase activity.

As presented in Figure 2D, KEGG pathway enrichment analysis of the 125 overlapping targets of OCEE in fracture treatment revealed the top 30 signaling pathways (*q* value < 0.05). These included nitrogen metabolism, IL‐17 signaling, bladder cancer, lipid metabolism and atherosclerosis, arachidonic acid (AA) metabolism, neural signal transmission, prostate cancer, the AGE‐RAGE signaling pathway in diabetes complications, chemically induced carcinogenesis—receptor activation, and ovarian steroidogenesis.

#### 3.2.4 “Drug Compounds‐Target‐Pathway” Interaction Network

**FIGURE 3:**
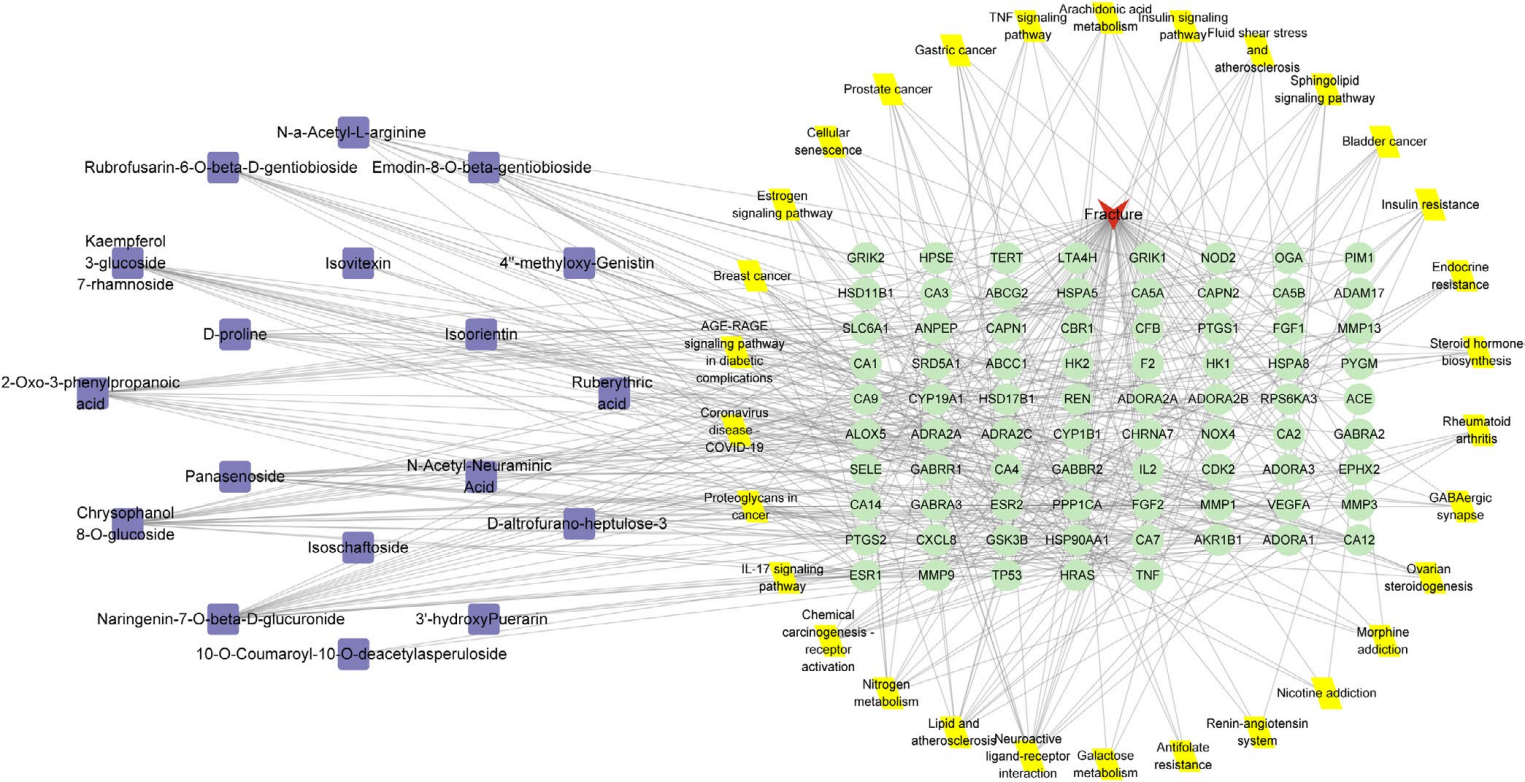
Visualization analysis of the “drug compounds‐target‐pathway” network. The red inverted triangle represents fracture, green circles represent targets, yellow parallelograms represent signaling pathways, and purple squares represent compounds.

A comprehensive “drug compounds‐target‐pathway” network model was developed, as shown in Figure 3. This model consists of 126 nodes, including 19 compound nodes, 77 target nodes, and 20 pathway nodes, highlighting the network's complexity. The network's topological parameters were examined utilizing the Network Analyzer plugin within Cytoscape software. The analysis demonstrated an average of 6.814 neighboring nodes, a network heterogeneity index of 1.0565, a network density of 0.055, and a centralization measure of 0.570. Based on Degree values (Table 4), the top five compounds were determined to be naringenin‐7‐O‐glucuronide (Degree 20), Chrysophanol‐8‐O‐glucoside (Degree 19), and 2‐Oxo‐3‐phenylpropanoic acid (Degree 17). The MS/MS spectra of these representative compounds were provided in Figure S1.

**TABLE 4:** Top 5 active compounds in OCEE with potential efficacy against fractures.

No.	Compound	Degree	BC	CC
1	Naringenin‐7‐O‐beta‐D‐glucuronide	20	0.674670	0.722543
2	Chrysophanol 8‐O‐glucoside	19	0.030219	0.396825
3	2‐Oxo‐3‐phenylpropanoic acid	17	0.023400	0.391850
4	Kaempferol 3‐glucoside 7‐rhamnoside	15	0.018371	0.384615
5	Panasenoside	14	0.016526	0.396825

### 3.3 MD Results

Following a comprehensive topological analysis, the top three core compounds, distinguished by their Degree values, were identified as Naringenin‐7‐O‐beta‐D‐glucuronide, Chrysophanol‐8‐O‐glucoside, and 2‐Oxo‐3‐phenylpropanoic acid. These core compounds were docked with the top five identified targets: GAPDH, TNF, TP53, MMP9, and ESR1, to calculate the binding energy (BE) and examine the interaction dynamics between the compounds and their respective targets. A BE value below zero signifies spontaneous binding, where more negative values indicate enhanced binding stability. As presented in Table 5, findings demonstrated that all three core compounds exhibited spontaneous binding to the five key targets. According to the literature (Hsin et al. 2013), 12 groups with BE < −5 kcal/mol exhibited strong binding, while 6 groups with BE < −7.0 kcal/mol displayed particularly intense binding. Among the core compounds, Naringenin‐7‐O‐beta‐D‐glucuronide demonstrated the strongest binding affinity with MMP9.

**TABLE 5:** Predicted binding energies of three potential active compounds to core targets combined with positive controls.

Targets (PDB ID)	Compounds	Binding energy (kcal·mol^−1^)
GAPDH (6 M61)	Naringenin‐7‐O‐beta‐D‐glucuronide	−7.48
	Chrysophanol 8‐O‐glucoside	−7.40
	2‐Oxo‐3‐phenylpropanoic acid	−4.87
	3‐Bromopyruvate^a^	−4.45
TNF (1TNF)	Naringenin‐7‐O‐beta‐D‐glucuronide	−7.67
	Chrysophanol 8‐O‐glucoside	−5.98
	2‐Oxo‐3‐phenylpropanoic acid	−4.66
	Thalidomide^a^	−4.98
TP53 (2BIM)	Naringenin‐7‐O‐beta‐D‐glucuronide	−6.99
	Chrysophanol 8‐O‐glucoside	−6.26
	2‐Oxo‐3‐phenylpropanoic acid	−4.86
	Nutlin‐3a^a^	−6.30
MMP9 (1GKC)	Naringenin‐7‐O‐beta‐D‐glucuronide	−7.96
	Chrysophanol 8‐O‐glucoside	−6.68
	2‐Oxo‐3‐phenylpropanoic acid	−5.73
	Ilomastat^a^	−6.05
ESR1 (1A52)	Naringenin‐7‐O‐beta‐D‐glucuronide	−7.50
	Chrysophanol 8‐O‐glucoside	−7.72
	2‐Oxo‐3‐phenylpropanoic acid	−5.02
	Tamoxifen^a^	−6.27

^a^ Target Inhibitors.

**FIGURE 4:**
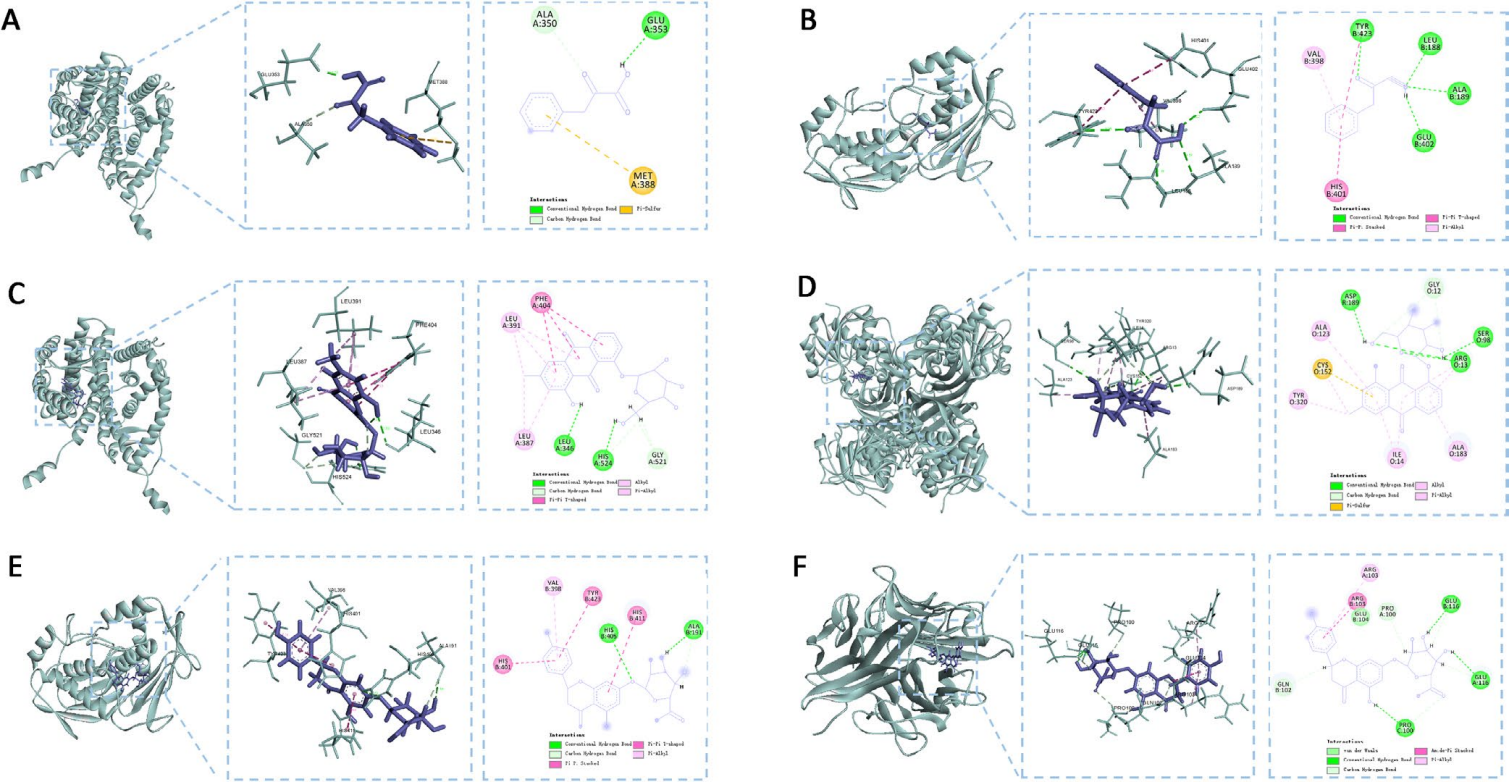
MD diagram. (A) 2‐Oxo‐3‐phenylpropanoic acid and ESR1; (B) 2‐Oxo‐3‐phenylpropanoic acid and MMP9; (C) Chrysophanol‐8‐*O*‐glucoside and ESR1; (D) Chrysophanol‐8‐*O*‐glucoside and GAPDH; (E) Naringenin‐7‐*O*‐beta‐D‐glucuronide and MMP9; (F) Naringenin‐7‐*O*‐beta‐D‐glucuronide and TNF.

The docking conformations with the highest BE between the core compounds and their respective targets are illustrated in Figure 4. As shown in Figure 4A, 2‐Oxo‐3‐phenylpropanoic acid established an H‐bond with ESR1 at GLU A:353, yielding a BE of −5.02 kcal/mol and indicating a strong interaction. In Figure 4B, 2‐Oxo‐3‐phenylpropanoic acid formed multiple H‐bonds with MMP9 at TYR B:423, LEU B:188, ALA B:189, and GLU B:402, with a BE of −5.73 kcal/mol, reflecting notable interaction strength. Figure 4C demonstrates that Chrysophanol‐8‐O‐glucoside formed interactions with ESR1 via H‐bonds at LEU A:346 and HIS A:524, achieving a BE of −7.72 kcal/mol and signifying a potent interaction. As presented in Figure 4D, Chrysophanol‐8‐O‐glucoside exhibited substantial H‐bond formation with GAPDH at SER O:98, ARG O:13, and ASP R:189, with a BE of −7.40 kcal/mol, suggesting a strong interaction. Figure 4E demonstrates that Naringenin‐7‐O‐beta‐D‐glucuronide established H‐bonds with MMP9 at HIS B:405 and ALA B:191, producing a BE of −7.96 kcal/mol and suggesting a robust interaction. Finally, Figure 4F shows that Naringenin‐7‐O‐beta‐D‐glucuronide formed H‐bonds with TNF at GLU B:116, GLU A:116, and PRO C:100, with a BE of −7.67 kcal/mol, reflecting a considerable interaction.

### 3.4 MDS Results

**FIGURE 5:**
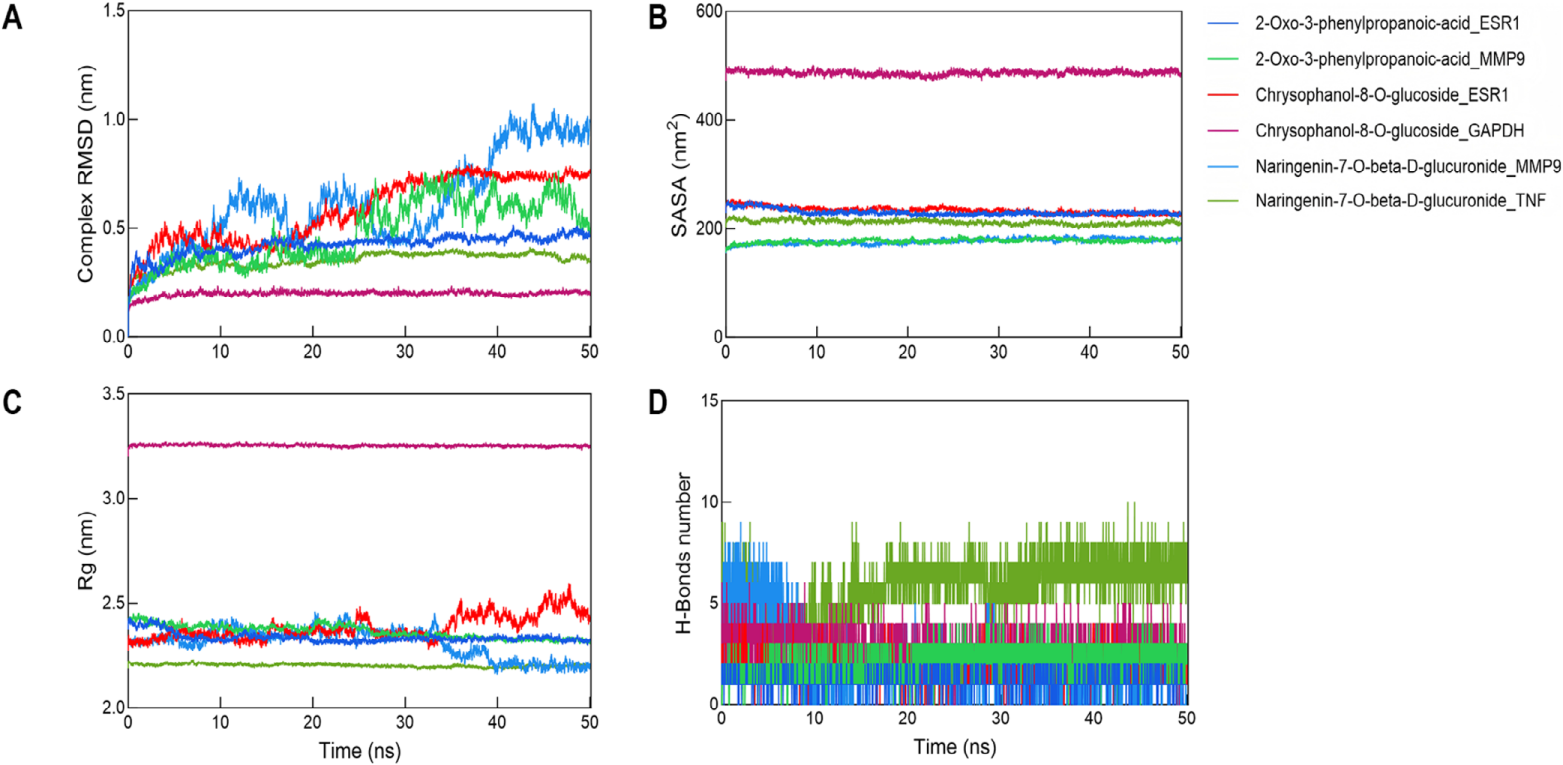
MDS results for the six complexes. (A) RMSD curve. (B) SASA curve. (C) Rg curve. (D) Number of hydrogen bonds.

Per the MD results, 50 ns MDS were executed to evaluate protein–ligand complex dynamic stability. RMSD was applied to assess complex stability, where reduced RMSD values indicated enhanced structural stability between protein and ligand (He et al. 2023). As shown in Figure 5A, the RMSD of Chrysophanol‐8‐O‐glucoside_ESR1 fluctuated slightly during the first 20 ns, followed by a gradual increase, suggesting that the complex's conformation adjusted during the latter part of the simulation. In contrast, 2‐Oxo‐3‐phenylpropanoic‐acid_ESR1, Chrysophanol‐8‐O‐glucoside_GAPDH, and Naringenin‐7‐O‐beta‐D‐glucuronide_TNF maintained relatively stable RMSD values throughout the simulation, indicating stable binding between the ligands and their respective proteins. However, the RMSD values of 2‐Oxo‐3‐phenylpropanoic‐acid_MMP9 and Naringenin‐7‐O‐beta‐D‐glucuronide_MMP9 fluctuated significantly, suggesting that the ligands in these two complexes may have undergone notable conformational changes.

SASA is a key indicator of the degree of exposure of hydrophobic and hydrophilic residues to the surrounding solvent (Richmond 1984). The results showed that Chrysophanol‐8‐O‐glucoside_GAPDH exhibited a relatively high SASA value, indicating that the protein in this complex might adopt a more open conformation. The SASA values for the other complexes remained relatively stable, suggesting good structural stability (Figure 5B). As shown in Figure 5C, the Rg of most complexes remained within the range of 2.2–2.5 nm, indicating that the protein structures of these complexes were generally stable. Notably, the Rg curve of 2‐Oxo‐3‐phenylpropanoic‐acid_MMP9 increased sharply at approximately 35 ns and exceeded 2.5 nm, while the Rg curve of Naringenin‐7‐O‐beta‐D‐glucuronide_MMP9 decreased suddenly at the same time point. Furthermore, Chrysophanol‐8‐O‐glucoside_GAPDH showed a relatively high Rg value, suggesting that the protein conformation in this complex may have expanded to a certain extent.

As presented in Figure 5D, Naringenin‐7‐O‐beta‐D‐glucuronide_TNF exhibited a relatively high number of H‐bonds, implying a potentially stronger binding interaction with the protein. The number of H‐bonds in the other complexes remained between 3 and 9, indicating relatively stable interactions between the ligands and proteins.

**FIGURE 6:**
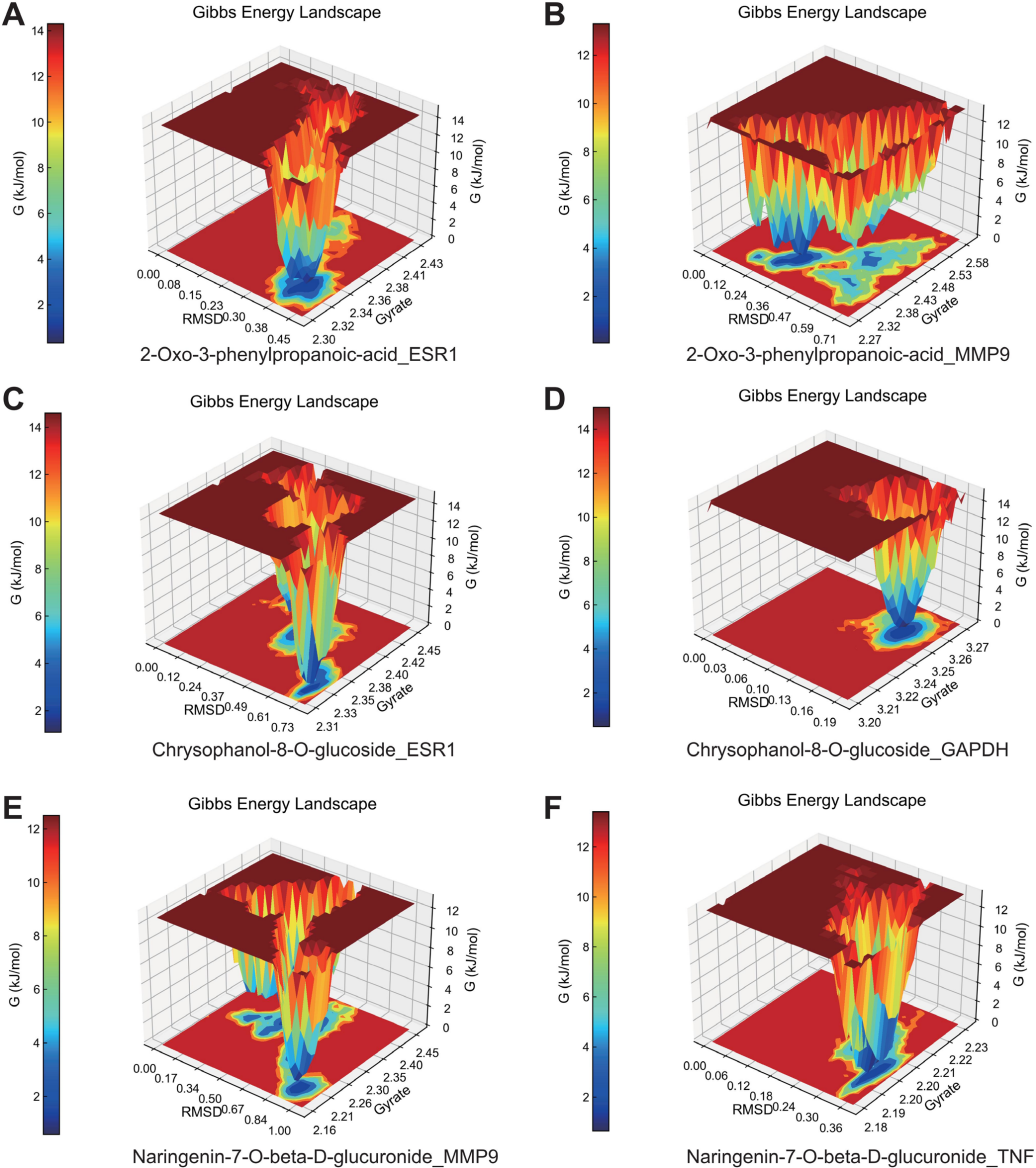
Gibbs energy analysis of six groups of complexes.

The Gibbs free energy profiles for the six complexes are depicted in Figure 6. The energy landscape of the 2‐Oxo‐3‐phenylpropanoic‐acid_ESR1 system reveals distinct low‐energy wells, indicating that the complex remains stable after ligand binding to the ESR1 protein (Figure 6A). In contrast, the energy landscape of the 2‐Oxo‐3‐phenylpropanoic‐acid_MMP9 system is more complex (Figure 6B), suggesting that the ligand undergoes more conformational changes within the MMP9 protein. Furthermore, the dispersed distribution of low free energy regions indicates the potential existence of multiple stable conformations in this system. For the Chrysophanol‐8‐O‐glucoside_ESR1 system (Figure 6C), the low free energy regions are concentrated, indicating a stable binding state between the ligand and the ESR1 protein. This finding aligns with the stability noted in the earlier RMSD analysis. The low free energy regions of the Chrysophanol‐8‐O‐glucoside_GAPDH system (Figure 6D) exhibit a narrow distribution, suggesting that the ligand adopts relatively stable conformations within the binding pocket of GAPDH, though with potentially fewer binding modes. Additionally, the GAPDH protein maintains a large Rg value after ligand binding, which may be related to its inherently open structural feature. The energy landscape of the Naringenin‐7‐O‐beta‐D‐glucuronide_MMP9 system (Figure 6E) displays multiple low‐energy wells, indicating that the ligand may bind to the MMP9 protein in various conformations. The presence of multiple stable regions suggests that the ligand possesses a degree of binding plasticity. Lastly, the energy landscape of the Naringenin‐7‐O‐beta‐D‐glucuronide_TNF system (Figure 6F) shows stable low‐energy regions, indicating that the ligand forms a small number of stable conformations in the TNF protein.

### 3.5 OCEE Enhances hFOB1.19 Cell Proliferation

**FIGURE 7:**
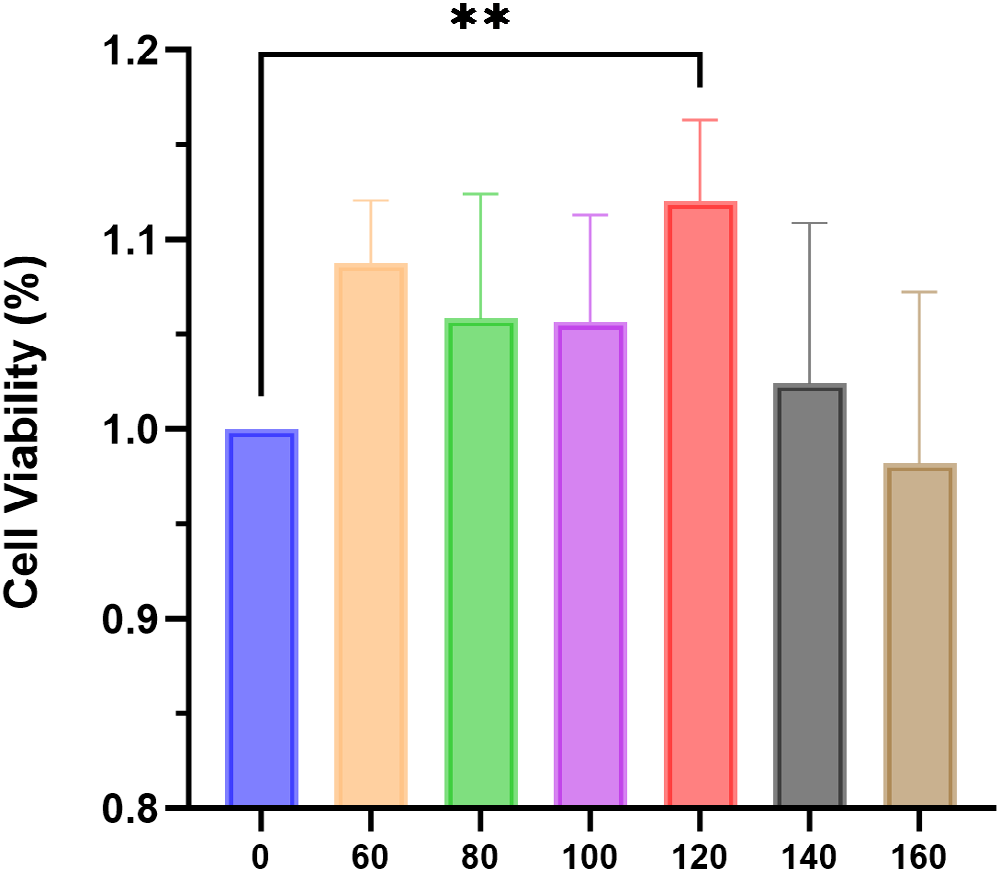
Influence of OCEE on hFOB1.19 cell proliferation. Data are presented as mean ± SD (*n* = 6 per group). Statistical analysis was performed using the Kruskal–Wallis test followed by Dunn's multiple comparisons test. ** indicates *p* < 0.01.

After 48‐h OCEE treatment, hFOB1.19 cells demonstrated increased proliferation (Figure 7). When exposed to 120 μg/mL concentration, the hFOB1.19 cell proliferation rate achieved its maximum level, markedly exceeding the control group (*p* < 0.05). Nevertheless, with a further elevation in OCEE concentration, the hFOB1.19 cell proliferation rate decreased.

### 3.6 OCEE Modulates Gene Expression in hFOB1.19 Cells

**FIGURE 8:**
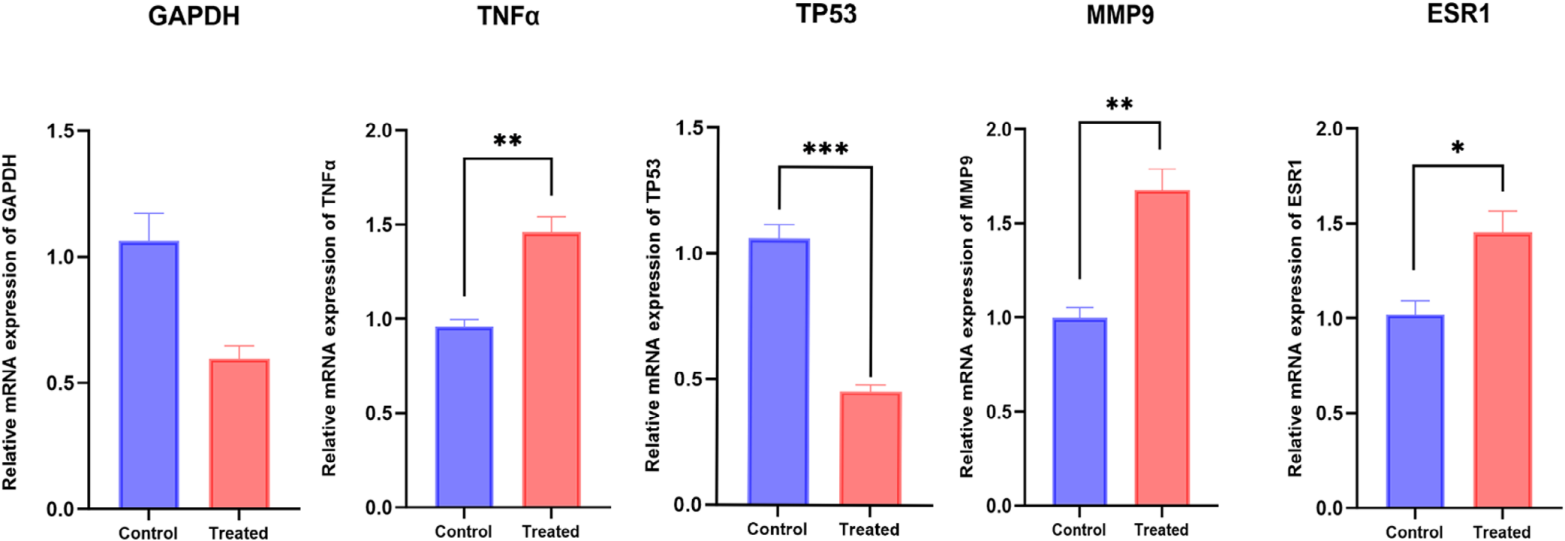
Modulation of fracture‐related gene expression by OCEE. Data are presented as mean ± SD (*n* = 3 per group). Statistical analysis was performed using the unpaired Student's *t*‐test (for normally distributed genes) or the Mann–Whitney *U* test (for GAPDH). * indicates *p* < 0.05, ** indicates *p* < 0.01, *** indicates *p* < 0.001 vs. the control group.

To confirm OCEE's regulatory effect on core fracture‐related targets, the expression of the top five identified targets was assessed using qPCR in hFOB1.19 cells (Figure 8). Treatment with 120 μg/mL OCEE for 48 h led to a marked elevation in TNFα, MMP9, and ESR1 expression levels versus the control group. Conversely, TP53 expression levels showed a significant reduction.

## 4 Discussion

Utilizing UHPLC‐Q‐Exactive‐MS/MS technology, this investigation successfully characterized 549 chemical compounds within OCEE. The top 20 compounds, identified by their relative content, were predominantly flavonoids and anthracenes, highlighting their dominance. Predictive analyses of the chemical compound targets of OCEE, as well as fracture‐related targets, revealed significant intersections, pinpointing naringenin‐7‐O‐beta‐D‐glucuronide and chrysophanol 8‐O‐glucoside as the most promising compounds for therapeutic efficacy. Dall'Asta et al. (2013) reported that naringenin‐7‐O‐β‐D‐glucuronide, a phase II conjugate metabolite of naringenin, is a flavonoid renowned for its broad pharmacological properties, including antioxidant, anti‐inflammatory, anticancer, and antibacterial activities, as well as cholesterol‐lowering effects (Akamo et al. 2021). Chrysophanol‐8‐O‐glucoside, a conjugated anthraquinone glycoside consisting of chrysophanol and glucose, demonstrates notable antibacterial (Janeczko et al. 2017), anti‐inflammatory, anticoagulant (Semwal et al. 2021), and cancer cell inhibitory properties (Wang et al. 2022), as well as a capacity to antagonize platelet aggregation, with a potency 5.45 times greater than aspirin (Tan et al. 2018). In MD analyses, both compounds demonstrated BE below −5 kcal/mol, indicating strong binding affinities to their respective targets. These activities are consistent with the regulatory demands of inflammatory response and tissue repair during fracture healing.

The PPI network analysis showed GAPDH, TNF, and TP53 as central targets for OCEE in fracture treatment, determined by their elevated node Degree values and linkages to numerous active compounds. GAPDH, essential to glycolysis, serves critical functions in cell proliferation (Sirover 2021) and apoptosis regulation (Sen et al. 2009). TNF, encoding a pro‐inflammatory cytokine, is implicated in various diseases, including autoimmune conditions, insulin resistance, psoriasis, and rheumatoid arthritis (Nie et al. 2013). Notably, TNF also serves as a key mediator controlling the inflammatory process during fracture healing. TP53, a crucial tumor suppressor gene, participates in DNA repair, cell cycle control, apoptosis, and differentiation (Voskarides and Giannopoulou 2023). These observations suggest that OCEE may exert its anti‐inflammatory effects through interactions with these identified targets. By comparing with previous studies, these hub targets further support that OCEE may improve fracture repair via regulating inflammatory and cellular processes.

Enrichment analysis demonstrated that the core targets participate in a network of 30 signaling pathways, including key routes such as nitrogen metabolism, IL‐17 signaling, and AA metabolism. In the nitrogen metabolism pathway, 10 carbonic anhydrase genes, including CA9, CA7, and CA12, play significant roles. These genes catalyze the reversible conversion of carbon dioxide and water into free carbonate ions (bicarbonate and hydrogen ions), thus actively contributing to the maintenance of cellular pH homeostasis (Supuran and Capasso 2020). IL‐17, a multifunctional pro‐inflammatory cytokine, is frequently associated with target genes that participate in antimicrobial activity and cytokine production, as confirmed by previous research (Tsai et al. 2013). The AA metabolism pathway has been well‐established as integral to the initiation and resolution of inflammatory responses (Wang et al. 2019). AA, a major component of cell membrane lipids, serves a vital function in lipid bilayer architecture (Koletzko and Decsi 1997). Its metabolism is mainly regulated by cyclooxygenase, lipoxygenase, and cytochrome P450 (CYP450) enzymes, which convert AA into a variety of metabolites, triggering an inflammatory cascade. AA is converted into 5‐hydroxyeicosatetraenoic acid and leukotrienes via 5‐lipoxygenase. Leukotrienes are potent inflammatory mediators that induce vasodilation, increase vascular permeability, and promote the adhesion and migration of inflammatory cells (Rubinstein and Dvash 2018). It is well documented that fracture healing undergoes a dual phase of inflammation: an early pro‐inflammatory phase and a late anti‐inflammatory or resolution phase. In this investigation, the IL‐17 pathway encompasses nine primary gene targets, including TNF, HSP90AA1, and MMP13, which serve crucial functions in host defense, tissue regeneration, inflammatory disease pathogenesis, and cancer progression. Collectively, these findings suggest that OCEE may regulate the balance between pro‐inflammatory and anti‐inflammatory phases during fracture healing, likely via modulation of the IL‐17 and AA metabolism pathways. Consistent with our network pharmacology predictions, qPCR validation confirmed that OCEE significantly upregulated the mRNA expression of TNFα, MMP9, and ESR1 in hFOB1.19 cells. TNFα is a core pro‐inflammatory cytokine that initiates the fracture repair cascade; TP53 regulates osteoblast proliferation and apoptosis, which is essential for bone callus formation; MMP9 mediates extracellular matrix remodeling and vascular invasion during fracture healing; and ESR1 promotes osteogenic differentiation and bone formation. Together, these genes play critical roles in the inflammatory, proliferative, and remodeling phases of fracture repair.

As a medicinal and edible plant commonly found in everyday diets, *Oxalis corniculata* L. has attracted growing interest for its potential in functional food development. This study establishes a systematic analytical framework for the plant's edible extract, combining high‐throughput chemical profiling, in silico target prediction, and multi‐level experimental validation. The model not only enhances the understanding of the material basis for *Oxalis corniculata* L.'s fracture‐healing effects as a functional food resource but also provides a solid experimental foundation for its application in functional foods and nutritional interventions aimed at bone health. Several limitations exist, including the lack of protein‐level validation, the use of a single cell line, and a relatively small sample size. In addition, compound identification by UHPLC‐Q‐Exactive‐MS/MS was based on database matching without validation using authentic standards, which represents another limitation of this study. Future research will employ authentic standard compounds and nuclear magnetic resonance technology to improve identification accuracy, perform quantitative analysis of key active metabolites, and establish animal fracture models to verify the in vivo effects of OCEE on fracture healing.

## 5 Conclusion

This study identified 549 compounds in OCEE using UHPLC‐Q‐Exactive‐MS/MS, with naringenin‐7‐O‐glucuronide, chrysophanol‐8‐O‐glucoside, and 2‐oxo‐3‐phenylpropanoic acid as the core active components. Network pharmacology, in silico simulations, and in vitro cell experiments confirmed that OCEE modulates key targets such as GAPDH, TNF, and TP53 and regulates nitrogen metabolism, IL‐17, and AA metabolism pathways, all of which contribute to osteogenic processes and fracture healing in vitro. These findings provide valuable in vitro data for understanding the chemical basis and potential mechanism of OCEE in fracture healing. Future studies will focus on in vivo studies and isolation and identification of active compounds to further verify these results, providing a new experimental strategy and theoretical basis for the development of *Oxalis corniculata* L. as functional foods and nutritional interventions for bone health.

## Author Contributions

**Xuegang Luo:** supervision, funding acquisition. **Xiaoyan Long:** investigation, methodology, writing – review and editing. **Xiaorong Zhao:** formal analysis, methodology, writing – review and editing. **Jian Zhang:** conceptualization, data curation, investigation, resources, writing – original draft. **Wanyan Shen:** investigation, writing – original draft, supervision. **Yu Zhang:** writing – review and editing, funding acquisition.

## Funding

This study was supported by The Science and Technology Planning Project of Guizhou Province ([2024]YB091); General fund of Guizhou Academy of Agricultural Sciences ([2024]10); and Guizhou Academy of Agricultural Sciences, Research on Innovation and Efficient Key Technologies of Characteristic Crop Germplasm in Guizhou Hot Zone (Qiannongke Germplasm Resources [2024] No. 08).

## Conflicts of Interest

The authors declare no conflicts of interest.

## Supporting information


**Figure S1:** MS/MS spectra of three key compounds identified in OCEE. (A) Naringenin‐7‐O‐glucuronide (Rt = 8.36 min, precursor ion m/z 449.29, elemental composition C_21_H_21_O_11_^+^) acquired in positive ion mode with higher‐energy collisional dissociation (HCD@40.00). (B) Chrysophanol 8‐O‐glucoside (Rt = 8.85 min, precursor ion m/z 461.11, elemental composition C_21_H_21_O_9_^−^, RDB = 18.0) acquired in negative ion mode. (C) 2‐Oxo‐3‐phenylpropanoic acid (Rt = 6.51 min, precursor ion m/z 147.04, elemental composition C_9_H_7_O_2_^+^) acquired in positive ion mode.

**Table S1:** A total of chemical constituents in *Oxalis corniculata* L. ethanol extract.

## Data Availability

All relevant data for this study are included in the article/Supporting Information.
