# Supplementary material for: Oxalis corniculata L. Ethanol Extract Promotes Fracture Healing: Integrated Omics and Experimental Validation
Source: Food Sci Nutr. 2026 May 19;14(5):e71896. doi: 10.1002/fsn3.71896 (PMC13184995; doi:10.1002/fsn3.71896)
Supplement: Supplementary file 1 — Figure S1: MS/MS spectra of three key compounds identified in OCEE. (A) Naringenin‐7‐O‐glucuronide (Rt = 8.36 min, precursor ion m/z 449.29, elemental composition C21H21O11 +) acquired in positive ion mode with higher‐energy collisional dissociation (HCD@40.00). (B) Chrysophanol 8‐O‐glucoside (Rt = 8.85 min, precursor ion m/z 461.11, elemental composition C21H21O9 −, RDB = 18.0) acquired in negative ion mode. (C) 2‐Oxo‐3‐phenylpropanoic acid (Rt = 6.51 min, precursor ion m/z 147.04, elemental composition C9H7O2 +) acquired in positive ion mode. [file FSN3-14-e71896-s001.docx]

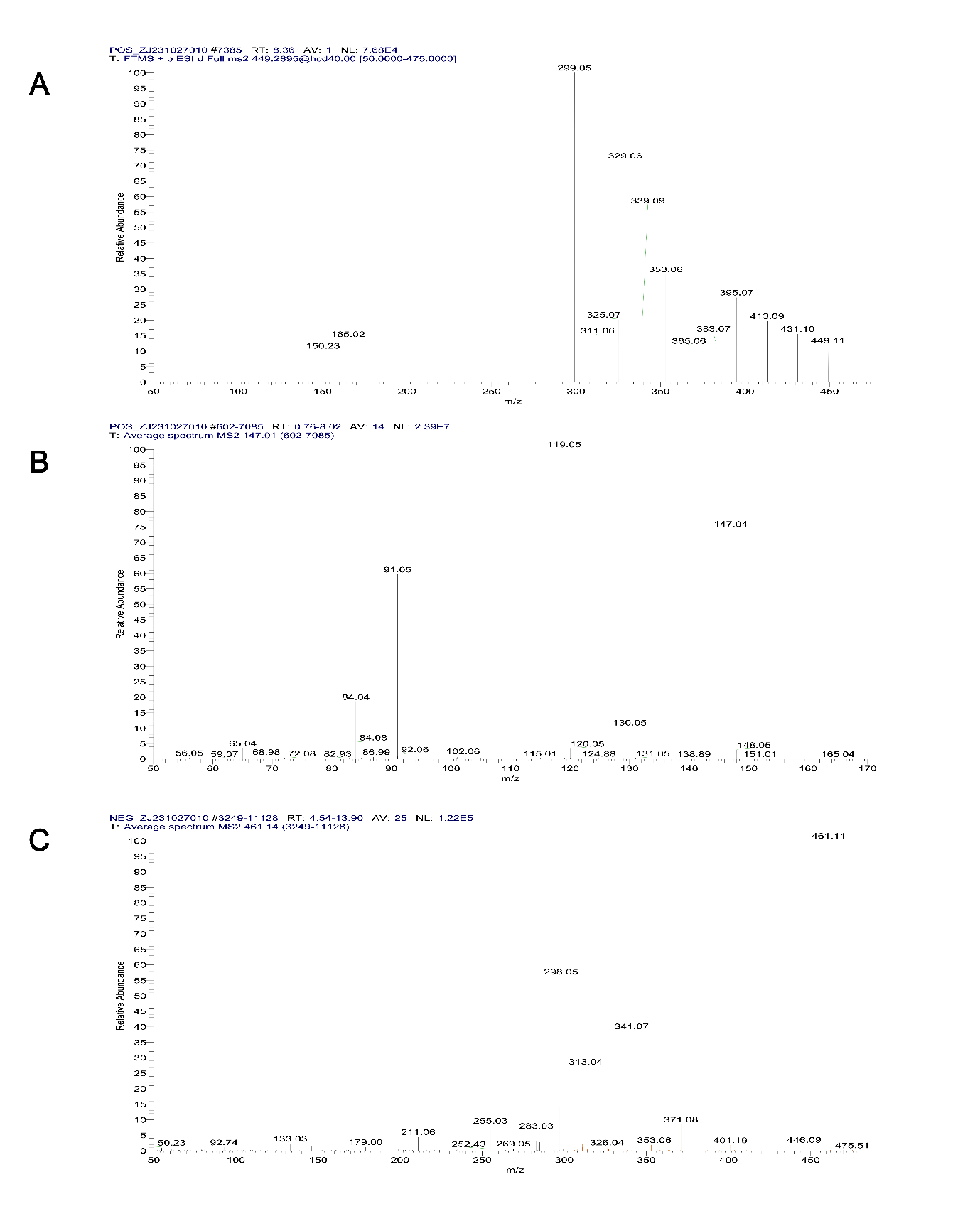


**Figure S1. MS/MS spectra of three key compounds identified in OCEE.** (A) Naringenin-7-O-glucuronide (Rt = 8.36 min, precursor ion m/z 449.29, elemental composition C₂₁H₂₁O₁₁⁺) acquired in positive ion mode with higher-energy collisional dissociation (HCD@40.00). (B) Chrysophanol 8-O-glucoside (Rt = 8.85 min, precursor ion m/z 461.11, elemental composition C₂₁H₂₁O₉⁻, RDB = 18.0) acquired in negative ion mode. (C) 2-Oxo-3-phenylpropanoic acid (Rt = 6.51 min, precursor ion m/z 147.04, elemental composition C₉H₇O₂⁺) acquired in positive ion mode.
