# Supplementary material for: Oxalis corniculata L. Ethanol Extract Promotes Fracture Healing: Integrated Omics and Experimental Validation
Source: Food Sci Nutr. 2026 May 19;14(5):e71896. doi: 10.1002/fsn3.71896 (PMC13184995; doi:10.1002/fsn3.71896)
Supplement: Supplementary file 2 — Table S1: A total of chemical constituents in Oxalis corniculata L. ethanol extract. [file FSN3-14-e71896-s002.docx]

**Table S1. A total of chemical constituents in *Oxalis corniculata* L. ethanol extract.**

| No. | Compound | Formula | Rt(min) | m/z | Adducts | Pubchem_ID |
| --- | --- | --- | --- | --- | --- | --- |
| 1 | 10-O-Coumaroyl-10-O-deacetylasperuloside | C_25_H_26_O_12_ | 8.04 | 563.1402503 | M+FA-H | 95224286 |
| 2 | 3'-hydroxyPuerarin | C_21_H_20_O_10_ | 8.75 | 433.1122012 | M+H, M+Na, 2M+Na, M+H-H_2_O, M+H-2H2O | 5748205 |
| 3 | Naringenin-7-O-beta-D-glucuronide | C_21_H_20_O_11_ | 8.23 | 449.1070109 | M+H-2H2O, M+H, M+Na, M+H-H2O | 15540754 |
| 4 | Isovitexin | C_21_H_20_O_10_ | 8.75 | 431.0978258 | M-H | 162350 |
| 5 | D-proline | C_5_H_9_NO_2_ | 1.03 | 116.0708423 | M+H, M+K, M+ACN+H, 2M+H | 8988 |
| 6 | Isoorientin | C_21_H_20_O_11_ | 8.23 | 447.0929221 | M-H2O-H, 2M-H, M-H | 114776 |
| 7 | 4''-methyloxy-Genistin | C_22_H_22_O_10_ | 8.82 | 445.1135254 | M-H, 2M-H | 71621984 |
| 8 | Isoschaftoside | C_26_H_28_O_14_ | 8.54 | 563.1402601 | M-H | 3084995 |
| 9 | Emodin-8-O-beta-gentiobioside | C_27_H_30_O_15_ | 7.60 | 593.1510053 | M-H | 71587230 |
| 10 | Betaine | C_5_H_11_NO_2_ | 0.94 | 118.0864485 | M+H | 247 |
| 11 | Kaempferol 3-glucoside 7-rhamnoside | C_27_H_30_O_15_ | 7.60 | 595.1649458 | M+H, M+Na, M+2Na-H, M+H-H2O | 57390614 |
| 12 | Ruberythric acid | C_25_H_26_O_13_ | 7.72 | 579.1352191 | M+FA-H | 92101 |
| 13 | N-Acetyl-Neuraminic Acid | C_11_H_19_NO_9_ | 1.67 | 290.0877245 | M-H2O-H, M-H | 439197 |
| 14 | Panasenoside | C_27_H_30_O_16_ | 7.24 | 611.1598517 | M+H, M+Na, M+H-H2O | 9986191 |
| 15 | 2-Oxo-3-phenylpropanoic acid | C_9_H_8_O_3_ | 6.51 | 147.0439352 | M+H-H2O | - |
| 16 | Chrysophanol 8-O-glucoside | C_21_H_20_O_9_ | 8.85 | 461.1085273 | M+FA-H | 442731 |
| 17 | N-a-Acetyl-L-arginine | C_8_H_16_N_4_O_3_ | 1.30 | 280.1384803 | M+H-2H2O, M+H, M+ACN+Na | 67427 |
| 18 | D-altrofurano-heptulose-3 | C_7_H_14_O_7_ | 0.95 | 245.0427951 | M+Cl | 145865405 |
| 19 | Trigonelline |  | 1.01 | 138.0549101 | M+? | 5570 |
| 20 | Rubrofusarin-6-O-beta-D-gentiobioside | C_27_H_32_O_15_ | 8.71 | 577.1559499 | M-H2O-H | 503733 |
| 21 | (-)-Epicatechin | C_15_H_14_O_6_ | 6.45 | 291.0856847 | M+H | 72276 |
| 22 | Verproside | C_22_H_26_O_13_ | 8.85 | 463.1225023 | M+H-2H2O | 12000799 |
| 23 | 4-AMINOBUTYRIC ACID | C_4_H_9_NO_2_ | 0.94 | 104.071033 | M+H-H2O, M+H | 119 |
| 24 | 5-Methoxypiperonal | C_9_H_8_O_4_ | 13.35 | 181.0493532 | M+H-H2O, M+H | 22016 |
| 25 | (+)-catechin | C_15_H_14_O_6_ | 6.45 | 289.0715174 | M-H | 9064 |
| 26 | Cholinesulfuric acid. | C_5_H_13_NO_4_S | 0.93 | 184.063628 | M+H | 485 |
| 27 | Neoschaftoside | C_26_H_28_O_14_ | 7.72 | 563.1402951 | M-H | 442619 |
| 28 | myo-Inositol | C_6_H_12_O_6_ | 1.06 | 179.0552031 | M-H | - |
| 29 | URIDINE | C_9_H_12_N_2_O_6_ | 2.79 | 243.0617864 | M+Cl, M+FA-H, M-H | 6029 |
| 30 | D-Glucosamine | C_6_H_13_NO_5_ | 0.96 | 180.0864432 | M+H, M+CH3OH+H | 439213 |
| 31 | D-Glutamic acid | C_5_H_9_NO_4_ | 0.93 | 148.0603084 | M+H | 23327 |
| 32 | Adenine | C_5_H_5_N_5_ | 1.30 | 136.0617794 | M+H | 190 |
| 33 | Sarracenin | C_11_H_14_O_5_ | 7.48 | 227.0908164 | M+H-H2O, M+H | - |
| 34 | Opuntiol | C_7_H_8_O_4_ | 1.06 | 174.0759588 | M+NH4 | 10034839 |
| 35 | 4-Hydroxycinnamic acid | C_9_H_8_O_3_ | 6.45 | 163.0391172 | M-H | 637542 |
| 36 | D-Valine | C_5_H_11_NO_2_ | 0.94 | 235.1647894 | 2M+H | 71563 |
| 37 | p-Vinylphenyl O-[beta-D-apiofuranosyl-(1-6)]-beta-D-glucopyranoside | C_19_H_26_O_10_ | 8.79 | 395.1341983 | M-H2O-H | 91895375 |
| 38 | D-LEUCINE | C_6_H_13_NO_2_ | 2.01 | 132.1019229 | M+H | 439524 |
| 39 | Lonfuranacid A | C_12_H_20_O_5_ | 9.90 | 243.1233044 | M-H, M+Na-2H | 146116229 |
| 40 | Gnetulin | C_30_H_26_O_8_ | 12.59 | 497.1585574 | M+H-H2O | 49843576 |
| 41 | Aurantiamide | C_25_H_26_N_2_O_3_ | 12.30 | 383.1760453 | M-H2O-H | 185904 |
| 42 | (E)-Cinnamyl Acetate | C_11_H_12_O_2_ | 12.29 | 194.1174013 | M+NH4, 2M+H, M+H | - |
| 43 | Afzelin | C_21_H_20_O_10_ | 10.05 | 431.0976996 | M-H | 5316673 |
| 44 | (_2_RS)-Lotaustralin | C_11_H_19_NO_6_ | 3.77 | 294.1539828 | M+CH3OH+H | 441467 |
| 45 | Carnitine | C_7_H_15_NO_3_ | 0.94 | 162.1122908 | M+H | 288 |
| 46 | Mercaptobenzothiazole | C_7_H_5_NS_2_ | 9.90 | 167.9935187 | M+H | 697993 |
| 47 | Guanosine | C_10_H_13_N_5_O_5_ | 2.05 | 284.098334 | M+H | 135398635 |
| 48 | L-glutamic acid | C_5_H_9_NO_4_ | 1.35 | 148.0603492 | M+H | 33032 |
| 49 | 2,7-Dimethyl-1,4-dihydroxynaphthalene 1-O-glucoside | C_18_H_22_O_7_ | 9.68 | 395.1340868 | M+FA-H | 134714923 |
| 50 | 4-Epialyxialactone | C_10_H_16_O_4_ | 10.08 | 199.0968504 | M-H, M+Na-2H | 14194344 |
| 51 | Noroxyhydrastinine | C_10_H_9_NO_3_ | 7.58 | 236.0559632 | M+FA-H | 89047 |
| 52 | 3-Methyladipic acid | C_7_H_12_O_4_ | 6.34 | 205.071081 | M+FA-H | 12292 |
| 53 | 4-Hydroxycinnamamide | C_9_H_9_NO_2_ | 6.18 | 198.03201 | M+Cl | 16637983 |
| 54 | Vitexin 2''-O-beta-D-glucoside | C_27_H_30_O_15_ | 7.28 | 595.1649996 | M+H | 5280641 |
| 55 | Enalin A | C_10_H_10_O_4_ | 6.81 | 177.054497 | M+H-H2O | 12084864 |
| 56 | p-Coumaric Acid Ethyl Ester | C_11_H_12_O_3_ | 10.86 | 191.0705473 | M-H | - |
| 57 | (E)-6-O-(p-coumaroyl)scandoside methyl ester | C_26_H_30_O_13_ | 7.06 | 595.1665991 | M+FA-H | 145874216 |
| 58 | 1-Linoleoyl-sn-glycero-3-phosphorylcholine | C_26_H_50_NO_7_P | 14.19 | 520.3388643 | M+H, M+Na | 11005824 |
| 59 | 4-Hydroxybenzoic acid | C_7_H_6_O_3_ | 6.70 | 137.0232978 | M-H | 135 |
| 60 | D-Glucosaminic acid | C_6_H_13_NO_6_ | 0.98 | 160.0603072 | M+H-2H2O | 73563 |
| 61 | 3,4-Dicaffeoylquinic acid | C_25_H_24_O_12_ | 8.94 | 515.1188567 | M-2H, M-H, M+Na-2H | 5281780 |
| 62 | Diacetylpiptocarphol | C_19_H_24_O_9_ | 9.68 | 397.1485048 | M+H, M+H-H2O | 102004563 |
| 63 | Methyl ferulate | C_11_H_12_O_4_ | 8.39 | 253.0713787 | M+FA-H | 5357283 |
| 64 | 3-O-Caffeoylquinic acid | C_16_H_18_O_9_ | 5.75 | 353.0874111 | M-H, M+Na-2H | 1794427 |
| 65 | Hispidulin 7-glucuronide | C_22_H_20_O_12_ | 8.44 | 475.0882097 | M-H | 5318059 |
| 66 | Parthenocissin A | C_28_H_22_O_6_ | 10.53 | 455.1479777 | M+H | - |
| 67 | Skimmin | C_15_H_16_O_8_ | 8.35 | 325.0909595 | M+H | 99693 |
| 68 | Syringic acid | C_9_H_10_O_5_ | 7.50 | 197.0448355 | M-H | 10742 |
| 69 | Perilloside B | C_16_H_24_O_7_ | 11.14 | 365.1024224 | M+K-2H | - |
| 70 | Syringin | C_17_H_24_O_9_ | 7.37 | 371.1342346 | M-H | 5316860 |
| 71 | Ampelopsin A | C_28_H_22_O_7_ | 11.48 | 453.1322504 | M+H-H2O | - |
| 72 | 7-Deoxyechinosporin | C_10_H_9_NO_4_ | 6.96 | 190.0497059 | M+H-H2O, M+NH4 | 11745823 |
| 73 | Lamiophlomiol A | C_11_H_14_O_6_ | 7.29 | 241.0713649 | M-H | 125923 |
| 74 | 1-Phenyl-2-propanol | C_9_H_12_O | 12.38 | 119.0857283 | M+H-H2O | 94185 |
| 75 | Chrysin 6-C-arabinoside 8-C-glucoside | C_26_H_28_O_13_ | 9.47 | 549.1592636 | M+H, M+Na | 21722007 |
| 76 | Virginiaebutanolide C | C_11_H_20_O_4_ | 11.23 | 215.1282735 | M-H, M+Na-2H | 40565489 |
| 77 | D-mannitol | C_6_H_14_O_6_ | 0.93 | 181.0708945 | M-H, M+FA-H, M-H2O-H | 6251 |
| 78 | Longicaulenone | C_12_H_18_O_4_ | 7.91 | 249.1108113 | M+Na | 25750965 |
| 79 | Asarylaldehyde | C_10_H_12_O_4_ | 7.28 | 197.0807442 | M+H-H2O, M+H | 20525 |
| 80 | Morroniside | C_17_H_26_O_11_ | 8.14 | 445.1120949 | M+K | 11228693 |
| 81 | D-Serine | C_3_H_7_NO_3_ | 0.90 | 147.0763052 | M+H-H2O, M+ACN+H | 71077 |
| 82 | Vanillic acid | C_8_H_8_O_4_ | 7.24 | 167.034072 | M-H | 8468 |
| 83 | Calystegine B_2_ | C_7_H_13_NO_4_ | 1.24 | 140.0705794 | M+H-2H2O | 124434 |
| 84 | prim-O-Glucosylangelicain | C_21_H_26_O_11_ | 8.88 | 499.1200849 | M+H-H2O, M+2Na-H | 91895378 |
| 85 | Ononin | C_22_H_22_O_9_ | 8.87 | 475.1239183 | M+FA-H | 442813 |
| 86 | Uracil | C_4_H_4_N_2_O_2_ | 2.87 | 113.034861 | M+H | 1174 |
| 87 | Quercimeritrin | C_21_H_20_O_12_ | 7.66 | 463.0878294 | M-H | 5282160 |
| 88 | Diosmetin-7-O-Beta-D-glucopyranoside | C_22_H_22_O_11_ | 9.37 | 463.1224977 | M+H, M+Na | 11016019 |
| 89 | LINAMARIN | C_10_H_17_NO_6_ | 1.30 | 289.1387787 | M+ACN+H | 11128 |
| 90 | P-Anisic acid | C_8_H_8_O_3_ | 7.56 | 151.039023 | M-H | - |
| 91 | 2-Ethyl-3-methylmaleimide N-alpha-D-glucopyranoside | C_13_H_19_NO_7_ | 0.88 | 319.1492812 | M+NH4 | - |
| 92 | 2-O-Methyluridine | C_10_H_14_N_2_O_6_ | 0.99 | 291.118058 | M+CH3OH+H | 102212 |
| 93 | Sec-O-Glucosylhamaudol | C_21_H_26_O_10_ | 9.30 | 439.1588931 | M+H, M+Na | 10478277 |
| 94 | ent-14,15-Dinor-13-oxolabda-8(17),11-dien-18-oic acid | C_18_H_26_O_3_ | 10.27 | 308.2212818 | M+NH4 | 91885074 |
| 95 | L-TYROSINE | C_9_H_11_NO_3_ | 2.17 | 180.0657163 | M-H | 6057 |
| 96 | Granatomycin E | C_22_H_22_O_11_ | 9.38 | 461.1085766 | M-H | 132331203 |
| 97 | Clostebol acetate | C_21_H_29_ClO_3_ | 1.08 | 401.1294477 | M+K-2H | 13327 |
| 98 | 2,6-Diaminoheptanedioic acid | C_7_H_14_N_2_O_4_ | 1.01 | 191.1023814 | M+H | 865 |
| 99 | Jaslanceoside B | C_26_H_30_O_14_ | 7.34 | 565.1558325 | M-H | 45359788 |
| 100 | Gaultherin | C_19_H_26_O_12_ | 8.28 | 485.1046449 | M+K | 5315244 |
| 101 | Ethyl alpha-D-ribo-hex-3-ulopyranoside | C_8_H_14_O_6_ | 6.82 | 171.0650882 | M+H-2H2O | - |
| 102 | 1-Oxo-4-hydroxy-2-en-4-ethylcyclohexa-5,8-olide | C_8_H_8_O_4_ | 2.82 | 186.0759291 | M+NH4 | 85844078 |
| 103 | Gallic acid | C_7_H_6_O_5_ | 3.11 | 169.0133166 | M-H | 370 |
| 104 | Mirificin | C_26_H_28_O_13_ | 9.48 | 547.1454426 | M-H | 21676217 |
| 105 | L-allo-Threonine | C_4_H_9_NO_3_ | 0.90 | 120.0656567 | M+H | 99289 |
| 106 | linoleic acid | C_18_H_32_O_2_ | 14.89 | 313.2729133 | M+CH3OH+H | 5280450 |
| 107 | Astraganoside | C_23_H_28_O_11_ | 10.77 | 481.1695093 | M+H, M+Na | 134715187 |
| 108 | 5-Hydroxy-2-methylchromone | C_10_H_8_O_3_ | 7.75 | 177.054491 | M+H | 821390 |
| 109 | Cleroindicin F | C_8_H_10_O_3_ | 6.50 | 119.0493656 | M+H-2H2O | 10374646 |
| 110 | 2-C-Methyl-D-erythrono-1,4-lactone | C_5_H_8_O_4_ | 1.26 | 133.0495657 | M+H, M+NH4 | 11126294 |
| 111 | L-Aspartic Acid 4-Benzyl Ester | C_11_H_13_NO_4_ | 4.87 | 222.0765745 | M-H | 101186 |
| 112 | Pedatisectine F | C_9_H_14_N_2_O_4_ | 3.52 | 215.1024092 | M+H | 12285902 |
| 113 | Axillarin | C_17_H_14_O_8_ | 8.23 | 327.0504732 | M-H2O-H | 5281603 |
| 114 | Xylitol | C_5_H_12_O_5_ | 0.94 | 151.0601245 | M-H | - |
| 115 | Lepidoside | C_26_H_28_O_14_ | 7.73 | 587.1362208 | M+Na | - |
| 116 | xanthosine | C_10_H_12_N_4_O_6_ | 4.20 | 283.0680089 | M-H | 64959 |
| 117 | Sterebin A | C_18_H_30_O_4_ | 11.81 | 309.2067099 | M-H | 21681091 |
| 118 | 3,7-Di-O-methylquercetin | C_17_H_14_O_7_ | 8.75 | 311.0556495 | M-H2O-H | 5280417 |
| 119 | Securiterpenoside | C_11_H_18_O_8_ | 1.15 | 296.1333534 | M+NH4 | 5321222 |
| 120 | Melittoside | C_21_H_32_O_15_ | 7.76 | 563.1387465 | M+K | 11968737 |
| 121 | L-Pyroglutamic acid | C_5_H_7_NO_3_ | 1.45 | 257.0774352 | 2M-H | 7405 |
| 122 | N-Acetyl-L-aspartic acid | C_6_H_9_NO_5_ | 1.28 | 158.0447045 | M+H-H2O | 65065 |
| 123 | 2-Adamantanone | C_10_H_14_O | 8.79 | 195.1019119 | M+FA-H | 64151 |
| 124 | visamminol-3'-O- glucoside | C_21_H_26_O_10_ | 9.86 | 437.1446502 | M-H | 163358503 |
| 125 | Acetyl-11-keto-Beta-boswellic acid | C_32_H_48_O_5_ | 12.60 | 513.3542283 | M+H | 9847548 |
| 126 | D-Aspartic acid | C_4_H_7_NO_4_ | 0.90 | 134.0447563 | M+H-H2O, M+H | 83887 |
| 127 | Eupatoriochromene | C_13_H_14_O_3_ | 9.03 | 219.1009084 | M+H | 100768 |
| 128 | Hydroxygenkwanin | C_16_H_12_O_6_ | 11.15 | 299.0556354 | M-H | 5318214 |
| 129 | 4'-Methoxypuerarin | C_22_H_22_O_9_ | 9.82 | 431.1326918 | M+H, M+Na | 5319486 |
| 130 | N-Acetylgalactosamine | C_8_H_15_NO_6_ | 0.92 | 266.0878365 | M+FA-H | 9800166 |
| 131 | N-Isobutyl-2,4,12-octadecatrienamide | C_22_H_39_NO | 12.91 | 378.2749826 | M+2Na-H | 25221579 |
| 132 | 8,9-Didehydro-7-hydroxydolichodial | C_10_H_12_O_3_ | 13.17 | 163.0751937 | M+H-H2O | 3062370 |
| 133 | Luteolin-3-O-beta-D-glucuronide | C_21_H_18_O_12_ | 8.87 | 461.0723816 | M-H | 10253785 |
| 134 | Aromadendrin 7-O-rhamnoside | C_21_H_22_O_10_ | 10.53 | 415.1029242 | M-H2O-H | 100927003 |
| 135 | Guanine | C_5_H_5_N_5_O | 1.88 | 150.0410516 | M-H, M+FA-H | 135398634 |
| 136 | Methyl gallate | C_8_H_8_O_5_ | 6.19 | 165.0183918 | M-H2O-H, M-H | 7428 |
| 137 | Cyclo(Tyr-Hpro) | C_14_H_16_N_2_O_4_ | 7.68 | 241.0968284 | M+H-2H2O | 102004913 |
| 138 | Sarmentosin | C_11_H_17_NO_7_ | 1.55 | 240.0862674 | M+H-2H2O | 5281123 |
| 139 | Clausine I | C_14_H_10_NO_3_ | 14.05 | 239.0591247 | M-H | - |
| 140 | Licofuranocoumarin | C_21_H_20_O_7_ | 9.83 | 429.1184585 | M+FA-H | 5319001 |
| 141 | 1,6-Dioxaspiro[4.5]decan-2-methanol | C_9_H_16_O_3_ | 8.65 | 217.1075444 | M+FA-H | 588029 |
| 142 | ADENOSINE | C_10_H_13_N_5_O_4_ | 4.06 | 268.1035088 | M+H | 60961 |
| 143 | Afzelechin 7-apioside | C_20_H_22_O_9_ | 9.86 | 439.1589343 | M+CH3OH+H | 13888255 |
| 144 | 2,6,6-Trimethyl-2,4-cycloheptadien-1-one | C_10_H_14_O | 9.30 | 183.1378461 | M+CH3OH+H | 136330 |
| 145 | Homononactinic acid | C_11_H_20_O_4_ | 9.77 | 181.1221661 | M+H-2H2O | 10889304 |
| 146 | 3-Hexen-1-ol O-b-D-glucopyranoside | C_12_H_22_O_6_ | 9.25 | 261.1340179 | M-H2O-H, M-H | 5318045 |
| 147 | Axillarin 4'-glucuronide | C_23_H_22_O_14_ | 7.48 | 503.0829582 | M-H2O-H | - |
| 148 | ETHYL CAFFEATE | C_11_H_12_O_4_ | 10.13 | 207.065604 | M-H | 5317238 |
| 149 | Pterodondiol | C_15_H_28_O_2_ | 13.50 | 285.2067125 | M+FA-H | 10879263 |
| 150 | Comanthosid B | C_23_H_22_O_12_ | 9.38 | 489.103358 | M-H | 38362596 |
| 151 | 4,5-Dihydroblumenol A | C_13_H_22_O_3_ | 10.24 | 191.1428456 | M+H-2H2O | 21630916 |
| 152 | 13-Hydroxygermacrone | C_15_H_22_O_2_ | 12.96 | 235.1689392 | M+H | 10399140 |
| 153 | 7,8-Dihydroxycoumarin | C_9_H_6_O_4_ | 7.13 | 223.0242731 | M-H, M+FA-H | 5280569 |
| 154 | Artemisinic acid | C_15_H_22_O_2_ | 12.17 | 235.1689544 | M+H, M+Na | 10922465 |
| 155 | Aconine | C_25_H_41_NO_9_ | 12.44 | 517.3129705 | M+NH4 | - |
| 156 | Scutellarin methyl ester | C_22_H_20_O_12_ | 7.37 | 475.0876461 | M-H | 14162695 |
| 157 | D-Arabinose | C_5_H_10_O_5_ | 0.95 | 133.0495368 | M+H-H2O | 854 |
| 158 | Durantoside I | C_26_H_32_O_13_ | 8.70 | 597.1825204 | M+FA-H | 95223135 |
| 159 | (-)-Epigallocatechin | C_15_H_14_O_7_ | 5.19 | 305.0662648 | M-H | 10425234 |
| 160 | Benzoylphenylalanine | C_16_H_15_NO_3_ | 10.55 | 268.0976227 | M-H | 97370 |
| 161 | 1,6-anhydro-b-D-Glucose | C_6_H_10_O_5_ | 1.23 | 207.0503356 | M+FA-H | 2724705 |
| 162 | Mogrol | C_30_H_52_O_4_ | 13.85 | 499.3749828 | M+Na | - |
| 163 | Vitexin-2''-O-p-trans-coumarate | C_30_H_26_O_12_ | 10.90 | 577.1347601 | M-H | 101422334 |
| 164 | Picrocrocin | C_16_H_26_O_7_ | 6.22 | 394.1850245 | M+ACN+Na | 130796 |
| 165 | Lamiidoside | C_26_H_32_O_14_ | 9.39 | 569.1854744 | M+H, M+Na | 23815404 |
| 166 | Acetylglycine | C_4_H_7_NO_3_ | 0.89 | 159.0762615 | M+ACN+H | 10972 |
| 167 | Secoxyloganin methyl ester | C_18_H_26_O_11_ | 8.14 | 383.1328993 | M+H-2H2O, M+K | 14105070 |
| 168 | Dihydrocarpanone | C_20_H_20_O_6_ | 14.19 | 357.1324383 | M+H | - |
| 169 | 1-Methylinosine | C_11_H_14_N_4_O_5_ | 7.84 | 317.0661774 | M+Cl | 65095 |
| 170 | Crotanecine | C_8_H_13_NO_3_ | 1.30 | 213.1231092 | M+ACN+H | 394146 |
| 171 | Cyclocerberidol | C_9_H_16_O_4_ | 7.17 | 153.0909433 | M+H-2H2O | 14466834 |
| 172 | Methyl jasminoside | C_27_H_32_O_13_ | 10.84 | 565.1906466 | M+H, M+Na | 45360098 |
| 173 | Cirsimaritin | C_17_H_14_O_6_ | 8.82 | 297.0746635 | M+H-H2O | 188323 |
| 174 | Monocillinol B | C_11_H_13_NO_5_ | 6.22 | 240.0857374 | M+H, M+Na | 10060146 |
| 175 | Plumieride | C_21_H_26_O_12_ | 8.86 | 491.1189187 | M+Na-2H | 72319 |
| 176 | docosapentaenoic acid | C_22_H_34_O_2_ | 13.62 | 375.2277517 | M+2Na-H | 5497182 |
| 177 | Cucumegastigmane I | C_13_H_20_O_4_ | 10.01 | 241.1430842 | M+H, M+NH4 | 16105430 |
| 178 | Jaceosidin | C_17_H_14_O_7_ | 11.13 | 329.0661884 | M-H | 5379096 |
| 179 | N-Acetyl-phenylalanine | C_11_H_13_NO_3_ | 8.27 | 206.0816227 | M-H | 74839 |
| 180 | Aurantio-obtusin Beta-D-glucoside | C_23_H_24_O_12_ | 9.37 | 493.1334118 | M+H, M+Na | 442725 |
| 181 | Chamaemeloside | C_27_H_28_O_14_ | 9.95 | 575.1403722 | M-H | - |
| 182 | Vanillin | C_8_H_8_O_3_ | 7.76 | 151.0390248 | M-H | 1183 |
| 183 | Kaempferol-3-O-galactoside | C_21_H_20_O_11_ | 9.08 | 447.0927824 | M-H | 5282149 |
| 184 | Inosine | C_10_H_12_N_4_O_5_ | 2.55 | 267.0731059 | M-H | 135398641 |
| 185 | Rosin | C_15_H_20_O_6_ | 5.52 | 314.1590299 | M+NH4 | 5280656 |
| 186 | Germanaism B | C_23_H_22_O_11_ | 7.58 | 475.1226659 | M+H-H2O, M+H | 57503926 |
| 187 | 4-Hydroxyphenylpyruvic acid | C_9_H_8_O_4_ | 5.85 | 163.0388536 | M+H-H2O | 979 |
| 188 | 13,14,15,16-Tetranor-8(17)-labden-12-oic acid | C_16_H_26_O_2_ | 12.53 | 295.190939 | M+FA-H | 14380023 |
| 189 | norepinephrine | C_8_H_11_NO_3_ | 4.58 | 214.0714572 | M+FA-H | 439260 |
| 190 | 5-Hydroxy-7-acetoxyflavone | C_17_H_12_O_5_ | 8.75 | 341.0660582 | M+FA-H | 5420895 |
| 191 | Gymnestrogenin | C_30_H_50_O_5_ | 13.81 | 513.3543437 | M+Na | 15560302 |
| 192 | Deoxyinosine | C_10_H_11_N_4_O_4_ | 4.45 | 269.1126039 | M+NH4 | 135398593 |
| 193 | 7-hydroxy-4-methyl-8-nitrocoumarin | C_10_H_7_NO_5_ | 0.84 | 242.006679 | M+Na-2H | 5376327 |
| 194 | Gamabufotalin | C_24_H_34_O_5_ | 12.24 | 403.2450024 | M+H | 259803 |
| 195 | caffeic acid | C_9_H_8_O_4_ | 6.80 | 163.0388641 | M+H-H2O | 689043 |
| 196 | 6-Demethoxycleomiscosin A | C_19_H_16_O_7_ | 9.24 | 401.087256 | M+FA-H | - |
| 197 | Kaempferitrin | C_27_H_30_O_14_ | 9.35 | 577.1559603 | M-H | 5486199 |
| 198 | Anonamine | C_19_H_28_NO_7_+ | 5.47 | 446.2010288 | M+ACN+Na | 6441178 |
| 199 | hypoxanthine | C_5_H_4_N_4_O | 4.26 | 137.0457796 | M+H | 135398638 |
| 200 | 2-Hydroxypalmitic acid | C_16_H_32_O_3_ | 14.74 | 271.2275341 | M-H | 92836 |
| 201 | Rengynic acid | C_8_H_14_O_4_ | 7.78 | 139.0753233 | M+H-2H2O, M+H, M+H-H2O | 54033324 |
| 202 | 3-Hydroxyperillaldehyde | C_10_H_14_O_2_ | 8.35 | 149.096055 | M+H-H2O | 85247137 |
| 203 | 6-O-Ethyltetradymodiol | C_17_H_26_O_3_ | 11.82 | 323.1854495 | M+FA-H | 162984443 |
| 204 | Senkyunolide I | C_12_H_16_O_4_ | 7.77 | 223.0970248 | M-H | 11521428 |
| 205 | Cynaroside | C_21_H_20_O_11_ | 9.84 | 447.0927666 | M-H | 5280637 |
| 206 | 5-deoxy Thymidine | C_10_H_14_N_2_O_4_ | 5.53 | 209.091829 | M+H-H2O | 65120 |
| 207 | Lupinol C | C_20_H_18_O_7_ | 8.82 | 393.0959145 | M+Na | 24094131 |
| 208 | 3,4-Dihydroxyphenylacetic acid | C_8_H_8_O_4_ | 5.09 | 167.0340605 | M-H | 547 |
| 209 | L-LYSINE | C_6_H_14_N_2_O_2_ | 0.77 | 147.1127112 | M+H | 5962 |
| 210 | N-(1-Carboxy-2-phenylethyl)glutamine | C_14_H_18_N_2_O_5_ | 6.53 | 293.1140428 | M-H | 558649 |
| 211 | Taxifolin-3-glucopyranoside | C_21_H_22_O_12_ | 9.52 | 449.1069226 | M+H-H2O | 3035567 |
| 212 | Cimifugin | C_16_H_18_O_6_ | 13.42 | 329.1012325 | M+Na | 441960 |
| 213 | Cleroindicin B | C_8_H_14_O_3_ | 6.68 | 141.0909611 | M+H-H2O | 184824 |
| 214 | 4-Oxododecanedioic acid | C_12_H_20_O_5_ | 10.02 | 225.1126602 | M-H2O-H | 13213508 |
| 215 | linolenic acid | C_18_H_30_O_2_ | 13.91 | 311.2573361 | M+CH3OH+H | 5280934 |
| 216 | Zeylenol | C_21_H_20_O_7_ | 10.08 | 349.1062293 | M+H-2H2O | 14283260 |
| 217 | Formononetin, 7-O-[-D-Apiofuranosyl-(16)--D-glu | C_27_H_30_O_13_ | 9.79 | 563.1746757 | M+H, M+Na | - |
| 218 | Furanofukinin | C_16_H_24_O_2_ | 12.19 | 293.1755262 | M+FA-H | 78385403 |
| 219 | 2-Hydroxy Hippuric Acid | C_9_H_9_NO_4_ | 4.37 | 213.0867448 | M+NH4 | 10253 |
| 220 | Juglanin | C_20_H_18_O_10_ | 8.69 | 417.0822596 | M-H | 5318717 |
| 221 | Scopoletin | C_10_H_8_O_4_ | 8.09 | 237.040001 | M+FA-H | 5280460 |
| 222 | luteolin | C_15_H_10_O_6_ | 10.55 | 287.0544553 | M+H | 5280445 |
| 223 | Quinic acid | C_7_H_12_O_6_ | 3.84 | 173.0446326 | M-H2O-H | 6508 |
| 224 | Genipin 1-alpha-D-gentiobioside | C_23_H_34_O_15_ | 7.10 | 573.1781479 | M+NH4, M+Na | - |
| 225 | N-Acetylleucine | C_8_H_15_NO_3_ | 7.92 | 174.1123903 | M+H, M+Na | 70912 |
| 226 | Furomollugin | C_14_H_10_O_4_ | 9.03 | 287.0557395 | M+FA-H | - |
| 227 | Scillascillin | C_17_H_12_O_6_ | 8.23 | 357.0610151 | M+FA-H | 75492722 |
| 228 | Eucamalol | C_10_H_16_O_2_ | 10.30 | 213.1125821 | M+FA-H | 12426239 |
| 229 | Isopentyl b-D-glucoside | C_11_H_22_O_6_ | 10.08 | 215.1275916 | M+H-2H2O | 10848285 |
| 230 | 8-Glucosyl-5,7-dihydroxy-2-isopropylchromone | C_18_H_22_O_9_ | 8.93 | 381.1186529 | M-H | 5317688 |
| 231 | 16-[(aminocarbonyl)hydrazono]stachan-18-oic acid | C_21_H_33_N_3_O_3_ | 14.54 | 376.2594678 | M+H | - |
| 232 | Resedine | C_9_H_9_NO_2_ | 8.62 | 146.0599762 | M+H-H2O | 202193 |
| 233 | ethyl 2-(1-hydroxy-4-oxocyclohexa-2,5-dien-1-yl)acetate | C_10_H_12_O_4_ | 7.24 | 195.0654442 | M-H | 100323 |
| 234 | Crotaleschenine | C_16_H_23_NO_5_ | 7.01 | 274.1432207 | M+H-2H2O | 21573658 |
| 235 | Wilforlide A | C_30_H_46_O_3_ | 14.66 | 455.349482 | M+H | - |
| 236 | 1-(4-Hydroxy-2,2-dimethylchroman-6-yl)ethanone | C_13_H_16_O_3_ | 12.72 | 221.1169591 | M+H-H2O, M+H | 129887823 |
| 237 | 2,4-Dihydroxy-6-methoxy-3-formylacetophenone | C_10_H_10_O_5_ | 6.28 | 255.0506395 | M+FA-H | 610933 |
| 238 | Isoboonein | C_9_H_14_O_3_ | 5.58 | 215.0918625 | M+FA-H | 10899112 |
| 239 | 1-Oleoyl-sn-glycero-3-phosphocholine | C_26_H_52_NO_7_P | 14.43 | 522.3540015 | M+H, M+Na | 16081932 |
| 240 | Qianhucoumarin A | C_19_H_20_O_6_ | 9.83 | 365.1023059 | M+Na-2H | 6444285 |
| 241 | Grasshopper ketone | C_13_H_20_O_3_ | 8.39 | 205.1227052 | M-H2O-H | 10220146 |
| 242 | 11-Hydroxyjasmonic acid | C_12_H_18_O_4_ | 9.25 | 259.1536033 | M+CH3OH+H | 15127090 |
| 243 | (-)-Gallocatechin | C_15_H_14_O_7_ | 5.20 | 307.0804549 | M+H | 9882981 |
| 244 | Rhodiocyanoside A | C_11_H_17_NO_6_ | 1.08 | 301.1387539 | M+ACN+H | 6442274 |
| 245 | Alisol F | C_30_H_48_O_5_ | 13.48 | 511.3390789 | M+Na | - |
| 246 | Enhydrin chlorohydrin | C_23_H_29_ClO_10_ | 11.53 | 481.1287911 | M-H2O-H | 91886687 |
| 247 | Kaempferol 3,4',7-triacetate | C_21_H_16_O_9_ | 8.84 | 445.1110167 | M+CH3OH+H | - |
| 248 | Alpinone 3-acetate | C_18_H_16_O_6_ | 12.74 | 329.1012353 | M+H | 15109631 |
| 249 | Jaborosalactone D | C_28_H_40_O_6_ | 10.89 | 490.3154205 | M+NH4 | 268947 |
| 250 | 8-O-Acetyltorilolone | C_17_H_26_O_4_ | 12.94 | 293.1755018 | M-H | 100932311 |
| 251 | Coniferaldehyde | C_10_H_10_O_3_ | 8.68 | 179.0701947 | M+H | 5280536 |
| 252 | Methyl 4-hydroxy-3-methoxycinnamate | C_11_H_12_O_4_ | 8.67 | 209.0806699 | M+H, M+Na | 5357283 |
| 253 | Linustatin | C_16_H_27_NO_11_ | 1.07 | 410.1646487 | M+H | 119301 |
| 254 | 3,7-Di-O-methylducheside A | C_22_H_20_O_12_ | 8.24 | 511.0633866 | M+Cl | - |
| 255 | Baicalin | C_21_H_18_O_11_ | 9.33 | 447.0913152 | M+H | 64982 |
| 256 | Regaloside C | C_18_H_24_O_11_ | 9.62 | 455.0939775 | M+K | 14135348 |
| 257 | Apigenin 7-O-methylglucuronide | C_22_H_20_O_11_ | 8.91 | 459.0929211 | M-H | 13844658 |
| 258 | 7-Aminocephalosporanic acid | C_10_H_12_N_2_O_5_S | 15.06 | 305.0799896 | M+CH3OH+H | - |
| 259 | Agnuside | C_22_H_26_O_11_ | 9.74 | 467.1538788 | M+H | 442416 |
| 260 | Protocatechualdehyde | C_7_H_6_O_3_ | 6.33 | 139.0388832 | M+H | 8768 |
| 261 | apigenin | C_15_H_10_O_5_ | 11.08 | 271.0596213 | M+H | 5280443 |
| 262 | Trifolirhizin | C_22_H_22_O_10_ | 8.13 | 469.1123032 | M+Na | 442827 |
| 263 | 20-Dehydroeupatoriopicrin semiacetal | C_20_H_24_O_6_ | 9.29 | 325.1427372 | M+H-2H2O | - |
| 264 | 3-O-Acetylpadmatin | C_18_H_16_O_8_ | 8.86 | 343.0799721 | M+H-H2O | 10406203 |
| 265 | Glycyrrhiza flavonol A | C_20_H_18_O_7_ | 9.35 | 415.102847 | M+FA-H | 5317765 |
| 266 | Lupinalbin A | C_15_H_8_O_6_ | 10.24 | 329.0298922 | M+FA-H | 5324349 |
| 267 | Cirsimarin | C_23_H_24_O_11_ | 8.86 | 459.1275946 | M+H-H2O | 159460 |
| 268 | Procyanidin B_3_ | C_30_H_26_O_12_ | 10.77 | 579.1487523 | M+H, M+Na | 146798 |
| 269 | Asperulosidic acid | C_18_H_24_O_12_ | 7.32 | 455.1151347 | M+Na | 11968867 |
| 270 | Glycolaldehyde dimer | C_4_H_8_O_4_ | 1.21 | 103.0394154 | M+H-H2O | 186078 |
| 271 | Cyanidin-3-O-glucoside chloride | C_21_H_21_ClO_11_ | 9.83 | 449.1068144 | M+? | 197081 |
| 272 | Chrysosplenetin | C_19_H_18_O_8_ | 12.46 | 373.0922493 | M-H | 5281608 |
| 273 | 1-(4-Hydroxybenzoyl)glucose | C_13_H_16_O_8_ | 4.61 | 345.0822314 | M+FA-H | 14132342 |
| 274 | ferulic acid | C_10_H_10_O_4_ | 9.59 | 193.0498695 | M-H | 445858 |
| 275 | Pinobanksin 3-(2-methyl)butyrate | C_20_H_20_O_6_ | 14.62 | 355.1181757 | M-H | 101190335 |
| 276 | 6(_1_H)-Azulenone, 2,3-dihydro-1,4-dimethyl | C_12_H_14_O | 10.48 | 219.1020113 | M+FA-H | 102004679 |
| 277 | Tricin 7-O-glucuronide | C_23_H_22_O_13_ | 8.90 | 505.0983449 | M-H | 101939793 |
| 278 | 3-Hydroxy-L-tyrosine | C_9_H_11_NO_4_ | 6.64 | 180.0653919 | M+H-H2O, M+Na | 6047 |
| 279 | Monocrotaline N-Oxide | C_16_H_23_NO_7_ | 7.68 | 364.135784 | M+Na | 197173 |
| 280 | Esculin | C_15_H_16_O_9_ | 6.27 | 339.071642 | M-H | 5281417 |
| 281 | Stearidonic acid | C_18_H_28_O_2_ | 13.81 | 321.2064931 | M+FA-H | 5312508 |
| 282 | Buspirone free base | C_21_H_31_N_5_O_2_ | 13.03 | 427.2808869 | M+ACN+H | 2477 |
| 283 | Deoxyguanosine | C_10_H_12_N_5_O_4_ | 5.69 | 267.0970661 | M+H | 135398592 |
| 284 | Eugenol rutinoside | C_22_H_32_O_11_ | 11.95 | 455.1930438 | M+H-H2O | 15101911 |
| 285 | 2,16-Kauranediol 2-O-beta-D-allopyranoside | C_26_H_44_O_7_ | 12.43 | 491.2970134 | M+Na | 73554066 |
| 286 | Europine N-oxide | C_16_H_27_NO_7_ | 8.67 | 346.1865323 | M+H | - |
| 287 | Cyclo(Tyr-Gly) | C_11_H_12_N_2_O_3_ | 5.04 | 221.0918048 | M+H | 138604 |
| 288 | 10-Gingerol | C_21_H_34_O_4_ | 12.83 | 373.2340932 | M+Na | 168115 |
| 289 | 1-(3,4,5-Trihydroxypentanoyl)-alpha-carboline | C_16_H_15_N_2_O_4_ | 6.89 | 363.1176636 | M+ACN+Na | - |
| 290 | [12]-Dehydrogingerdione | C_23_H_34_O_4_ | 11.76 | 438.2627821 | M+ACN+Na | 154791045 |
| 291 | Axillaridine | C_18_H_27_NO_6_ | 13.60 | 371.2170684 | M+NH4 | 179398 |
| 292 | Coixol | C_8_H_7_NO_3_ | 4.41 | 210.0401223 | M+FA-H | 10772 |
| 293 | Dimeric coniferyl acetate | C_24_H_26_O_8_ | 12.14 | 479.1130572 | M+K-2H | - |
| 294 | 2-Hydroxy-4-methoxybenzaldehyde | C_8_H_8_O_3_ | 6.72 | 185.0808469 | M+CH3OH+H | 69600 |
| 295 | Ethyl gallate | C_9_H_10_O_5_ | 7.98 | 197.0447901 | M-H | 13250 |
| 296 | Peujaponiside | C_25_H_34_O_14_ | 11.26 | 523.1802035 | M+H-2H2O | 197658 |
| 297 | 6-O-p-Methoxycinnamoylcatalpol | C_25_H_30_O_12_ | 11.24 | 545.1622248 | M+Na | 91895358 |
| 298 | ellagic acid | C_14_H_6_O_8_ | 9.37 | 300.9985756 | M-H | 5281855 |
| 299 | Sibiricose A_3_ | C_19_H_26_O_13_ | 9.58 | 501.0994776 | M+CH3OH+H, M+K | 10813903 |
| 300 | 27-O-acetyl-withaferin A | C_31_H_42_O_6_ | 12.16 | 552.3304524 | M+ACN+H, M+2Na-H | - |
| 301 | cafestol | C_20_H_28_O_3_ | 12.38 | 299.1974748 | M+H-H2O | 108052 |
| 302 | Methyl 2-(2-hydroxyphenyl)acetate | C_9_H_10_O_3_ | 8.08 | 208.0967118 | M+H-H2O, M+ACN+H | - |
| 303 | 3-Hydroxy-p-menth-1-en-6-one | C_10_H_16_O_2_ | 11.68 | 151.1116616 | M+H-H2O, M+H | 14106048 |
| 304 | L(+)-Ascorbic acid | C_6_H_8_O_6_ | 1.23 | 221.029659 | M+FA-H | 54670067 |
| 305 | Mandelic acid | C_8_H_8_O_3_ | 9.70 | 151.039006 | M-H | 1292 |
| 306 | Eupalitin 3-galactoside | C_23_H_24_O_12_ | 9.77 | 475.1224912 | M+H-H2O | 9805669 |
| 307 | trans-Dehydrocurvularin | C_16_H_18_O_5_ | 6.53 | 271.097345 | M-H2O-H | 6438143 |
| 308 | Suavioside A | C_26_H_44_O_8_ | 11.54 | 507.2921564 | M+Na | 73821014 |
| 309 | p-Hydroxybenzaldehyde | C_7_H_6_O_2_ | 7.29 | 123.0443077 | M+H | 126 |
| 310 | Procyanidin B_1_ | C_30_H_26_O_12_ | 6.54 | 577.1349514 | M-H | 11250133 |
| 311 | 6-O-Vanilloylajugol | C_23_H_30_O_12_ | 9.39 | 481.1695481 | M+H-H2O | 14396664 |
| 312 | Sugeroside | C_26_H_42_O_8_ | 11.74 | 505.2763971 | M+Na | 3082543 |
| 313 | 2-Pentadecenedioic acid | C_15_H_26_O_4_ | 12.52 | 251.164852 | M-H2O-H | 12867460 |
| 314 | Methyl hexadecanoate | C_17_H_34_O_2_ | 13.20 | 315.2534689 | M+FA-H | 8181 |
| 315 | Paeonilactone A | C_10_H_14_O_4_ | 6.75 | 216.1228289 | M+NH4 | 10081437 |
| 316 | 20(S)-Protopanaxadiol | C_30_H_52_O_3_ | 14.24 | 483.3800676 | M+Na | - |
| 317 | Myricananin A | C_20_H_24_O_5_ | 9.20 | 389.1334034 | M+2Na-H | 25141365 |
| 318 | Geniposide | C_17_H_24_O_10_ | 7.49 | 427.1000261 | M+K | 107848 |
| 319 | Indican | C_14_H_16_NO_6_ | 5.70 | 339.0679198 | M+2Na-H | 441564 |
| 320 | 17-Hydroxyisolathyrol | C_20_H_30_O_5_ | 11.77 | 349.1989547 | M-H | 127256191 |
| 321 | Salviaflaside | C_24_H_26_O_13_ | 8.95 | 545.1282676 | M+Na | 6438919 |
| 322 | Sanggenone K | C_30_H_32_O_6_ | 11.02 | 471.2190073 | M+H-H2O | 44258299 |
| 323 | Quinaldic acid | C_10_H_7_NO_2_ | 7.04 | 215.0813745 | M+ACN+H | 7124 |
| 324 | Niazirin | C_14_H_17_NO_5_ | 9.57 | 302.0992336 | M+H, M+Na | 129556 |
| 325 | Methoxyeugenol 4-O-rutinoside | C_23_H_34_O_12_ | 10.55 | 467.1901276 | M+H-2H2O | 102384367 |
| 326 | Jasminoside N | C_22_H_38_O_11_ | 10.60 | 501.2297026 | M+Na | 101505270 |
| 327 | falcarindiol | C_17_H_24_O_2_ | 12.59 | 261.1826524 | M+H | 5281148 |
| 328 | Eupatorin | C_18_H_16_O_7_ | 12.35 | 345.0963563 | M+H | 97214 |
| 329 | Dihydrolycorine | C_16_H_19_NO_4_ | 6.18 | 290.1379585 | M+H | - |
| 330 | Damnacanthol | C_16_H_12_O_5_ | 7.83 | 317.1013297 | M+CH3OH+H | - |
| 331 | Koenigine | C_19_H_18_NO_3_ | 9.85 | 329.1025746 | M+Na-2H | 5318825 |
| 332 | Piceoside | C_14_H_18_O_7_ | 9.00 | 281.101381 | M+H-H2O, M+Na | 92123 |
| 333 | 6-Shogaol | C_17_H_24_O_3_ | 12.58 | 259.1667357 | M+H-H2O | 5281794 |
| 334 | Shikimic Acid | C_7_H_10_O_5_ | 6.85 | 173.0446746 | M-H | 8742 |
| 335 | _7_alpha-Galloyloxysweroside | C_23_H_26_O_14_ | 7.23 | 491.1176425 | M+H-2H2O | - |
| 336 | trans-3-Indoleacrylic acid | C_11_H_8_NO_2_ | 8.68 | 207.0292687 | M+Na-2H | 5375048 |
| 337 | Dianthoside | C_12_H_16_O_8_ | 1.21 | 306.1176908 | M+NH4 | 5316639 |
| 338 | Ethyl vanillin acetate | C_11_H_12_O_4_ | 8.68 | 207.065621 | M-H | 155708 |
| 339 | (+)-Mellein | C_10_H_10_O_3_ | 8.68 | 177.054839 | M-H | 28516 |
| 340 | Diosmetin | C_16_H_12_O_6_ | 9.39 | 301.0696613 | M+H | 5281612 |
| 341 | 2''-O-Coumaroyljuglanin | C_29_H_24_O_12_ | 9.20 | 597.1589997 | M+CH3OH+H | 23265178 |
| 342 | Narchinol B | C_12_H_16_O_3_ | 8.77 | 191.1063624 | M+H-H2O | 56835095 |
| 343 | Spectabiline | C_18_H_25_NO_7_ | 4.96 | 332.1484162 | M+H-2H2O | 73414 |
| 344 | Syringaldehyde | C_9_H_10_O_4_ | 7.94 | 181.0498003 | M-H | 8655 |
| 345 | Odontoside | C_20_H_22_O_11_ | 9.44 | 403.1015309 | M+H-2H2O | 5320735 |
| 346 | trans-5-Hydroxyferulic acid | C_10_H_10_O_5_ | 7.79 | 209.0449332 | M-H | 446834 |
| 347 | 5-Hydroxy-6,7-dimethoxylflavone | C_17_H_14_O_5_ | 8.95 | 331.1169307 | M+CH3OH+H | 471722 |
| 348 | 5-Acetylsalicylic acid | C_9_H_8_O_4_ | 6.69 | 181.0494286 | M+H, M+Na | 83151 |
| 349 | Viscumneoside III | C_27_H_32_O_15_ | 8.69 | 561.1594412 | M+H-2H2O | 195287 |
| 350 | Diderroside | C_19_H_28_O_13_ | 8.94 | 501.1033954 | M+K-2H | 23760099 |
| 351 | Protoescigenin | C_30_H_50_O_6_ | 13.47 | 529.3494563 | M+Na | 15560300 |
| 352 | Arteannuin B | C_15_H_20_O_3_ | 4.13 | 271.1282698 | M+Na | 6543478 |
| 353 | Palatiferin A | C_21_H_20_O_8_ | 7.46 | 418.1483732 | M+NH4 | - |
| 354 | Alpinumisoflavone acetate | C_22_H_18_O_6_ | 12.24 | 359.0919653 | M-H2O-H | 5490139 |
| 355 | Isosclerone | C_10_H_10_O_3_ | 7.23 | 196.0967079 | M+NH4 | 13369486 |
| 356 | Santin | C_18_H_16_O_7_ | 12.18 | 345.0977696 | M+H | 5281695 |
| 357 | Phenylacetylglycine | C_10_H_11_NO_3_ | 8.11 | 174.0551525 | M-H2O-H | 68144 |
| 358 | Orcinol glucoside | C_13_H_18_O_7_ | 7.61 | 350.120188 | M+ACN+Na | - |
| 359 | 2-(-D-Glucopyranosyloxy)-4-hydroxybenzenepropanoi | C_15_H_20_O_9_ | 8.35 | 309.0961553 | M+H-2H2O | - |
| 360 | Damascenone | C_13_H_18_O | 10.51 | 173.1325087 | M+H-H2O | 5366074 |
| 361 | _5_alpha-Hydroxychloranthalactone A | C_15_H_16_O_3_ | 7.75 | 286.1430547 | M+ACN+H | 131857139 |
| 362 | resveratrol | C_14_H_12_O_3_ | 8.99 | 227.0707466 | M-H | 445154 |
| 363 | Iridin | C_24_H_26_O_13_ | 9.87 | 523.1437471 | M+H | 5281777 |
| 364 | Dihydrocitrinone | C_13_H_14_O_6_ | 7.25 | 330.0941728 | M+ACN+Na | 163095 |
| 365 | Nevadensin | C_18_H_16_O_7_ | 12.20 | 343.0817133 | M-H | 160921 |
| 366 | Genkwanin | C_16_H_12_O_5_ | 12.26 | 283.0607518 | M-H | 5281617 |
| 367 | Isosaxalin | C_16_H_16_O_6_ | 6.44 | 349.0923398 | M+FA-H | 511359 |
| 368 | Moracin P | C_19_H_18_O_5_ | 12.89 | 371.1139487 | M+FA-H | 25208124 |
| 369 | Albatrelin A | C_24_H_34_O_4_ | 11.66 | 404.2785016 | M+NH4 | 71524348 |
| 370 | Ethyl 3,4-dihydroxybenzoate | C_9_H_10_O_4_ | 9.20 | 181.0497772 | M-H | 77547 |
| 371 | Orcinol gentiobioside | C_19_H_28_O_12_ | 6.92 | 490.1907457 | M+ACN+H | 10411370 |
| 372 | Rhodiosin | C_27_H_30_O_16_ | 7.49 | 633.1414812 | M+Na | - |
| 373 | 4',5,7-Trihydroxy 3,3',6,8-tetramethoxyflavone | C_19_H_18_O_9_ | 7.60 | 355.0803271 | M+H-2H2O | 5386959 |
| 374 | Feruloylputrescine | C_14_H_20_N_2_O_3_ | 6.27 | 263.1397415 | M-H | 5281796 |
| 375 | Dihydroxyalnusone | C_19_H_18_O_5_ | 7.16 | 344.1485613 | M+NH4 | 101226713 |
| 376 | hesperetin | C_16_H_14_O_6_ | 5.72 | 303.0857651 | M+H | 72281 |
| 377 | VERATRIC ACID | C_9_H_10_O_4_ | 8.55 | 181.0498244 | M-H | 7121 |
| 378 | 4-Hydroxyproline | C_7_H_11_NO_4_ | 4.63 | 369.1283644 | 2M+Na | 825 |
| 379 | Zeatin | C_10_H_13_N_5_O | 6.61 | 264.0838325 | M+2Na-H | 449093 |
| 380 | Kushenol Q | C_25_H_30_O_7_ | 13.25 | 479.1495134 | M+K-2H | 42608036 |
| 381 | Oxychlororaphine | C_13_H_9_N_3_O | 12.04 | 268.0724194 | M+FA-H | 120282 |
| 382 | 1-Monolinolenin | C_21_H_36_O_4_ | 13.25 | 416.2760304 | M+ACN+Na | 5872092 |
| 383 | Quercetin 3-O-glucuronide | C_21_H_18_O_13_ | 9.04 | 477.066922 | M-H, M+Na-2H | NEG |
| 384 | Scutebarbatine Z | C_26_H_33_NO_5_ | 8.63 | 484.2067836 | M+2Na-H | 46929396 |
| 385 | Isoquercitrin | C_21_H_20_O_12_ | 9.01 | 463.0878386 | M-H | 5280804 |
| 386 | Pantoyllactone glucoside | C_12_H_20_O_8_ | 7.68 | 257.1014135 | M+H-2H2O | 14701560 |
| 387 | Pterisolic acid B | C_20_H_26_O_4_ | 10.20 | 348.2160823 | M+NH4 | 53238558 |
| 388 | _8_alpha-Hydroxyhirsutinolide | C_15_H_20_O_6_ | 12.11 | 261.1118324 | M+H-2H2O | 70690654 |
| 389 | Oridonin | C_20_H_28_O_6_ | 13.50 | 365.1929174 | M+H | - |
| 390 | Meridinol | C_20_H_18_O_7_ | 9.67 | 353.1011904 | M+H-H2O | 11079164 |
| 391 | Tetillapyrone | C_11_H_14_O_6_ | 1.56 | 260.1124279 | M+NH4 | 11010113 |
| 392 | Sesamoside | C_17_H_24_O_12_ | 5.73 | 443.115216 | M+Na | 3082856 |
| 393 | 3,7-O-Diacetylpinobanksin | C_19_H_16_O_7_ | 8.86 | 357.0959347 | M+H | 91884891 |
| 394 | Demethoxyencecalinol | C_13_H_16_O_2_ | 12.70 | 249.1127277 | M+FA-H | 156221 |
| 395 | Quercetin 3-O-beta-(6''-p-coumaroyl)glucopyranosyl(1->2)-alpha-L-rhamnopyranoside | C_36_H_36_O_18_ | 3.82 | 379.1033511 | M+2H | - |
| 396 | Artemitin | C_20_H_20_O_8_ | 8.41 | 389.1199899 | M+H | 5320351 |
| 397 | Gentiopicroside | C_16_H_20_O_9_ | 8.23 | 395.0753141 | M+K | 88708 |
| 398 | 1_1_alpha,1_2_alpha-Epoxy-_3_beta,23-dihydroxy-30-norolean-20(29)-en-28,1_3_beta-olide | C_29_H_42_O_6_ | 11.94 | 504.3310755 | M+NH4 | 70698158 |
| 399 | 3,19-Dihydroxy-6,23-dioxo-12-ursen-28-oic acid | C_30_H_44_O_6_ | 12.15 | 518.3466682 | M+NH4 | 15460490 |
| 400 | Gastrodin | C_13_H_18_O_7_ | 6.59 | 309.0939943 | M+Na | 115067 |
| 401 | Obscuraminol E | C_16_H_33_NO | 14.85 | 288.2890053 | M+CH3OH+H | 134715064 |
| 402 | Petiolin G | C_21_H_22_O_11_ | 9.87 | 433.1121608 | M+H-H2O | 44473623 |
| 403 | Hyperwightin C | C_20_H_22_O_10_ | 8.68 | 445.1120754 | M+Na | 168011860 |
| 404 | Monotropein | C_16_H_22_O_11_ | 6.28 | 413.1066872 | M+Na | 73466 |
| 405 | 5'-Methoxylariciresinol | C_21_H_26_O_7_ | 7.78 | 429.1327262 | M+K | 184458 |
| 406 | Asterbatanoside A | C_19_H_26_O_11_ | 7.84 | 411.1291143 | M-H2O-H | 127686 |
| 407 | N-Vanillyldecanamide | C_18_H_29_NO_3_ | 10.20 | 306.2070041 | M-H | 169252 |
| 408 | 4-Hydroxybenzaldehyde rhamnoside | C_13_H_16_O_6_ | 5.91 | 286.1278061 | M+NH4 | 11777785 |
| 409 | Syringolin A | C_24_H_39_N_5_O_6_ | 12.18 | 535.3237713 | M+ACN+H | 42601513 |
| 410 | 5,7-Dihydroxyphthalide | C_8_H_6_O_4_ | 6.15 | 211.0241969 | M+FA-H | 11062751 |
| 411 | Taiwapyrone | C_10_H_14_O_4_ | 8.95 | 163.0752543 | M+H-2H2O | 101316864 |
| 412 | Ruscogenin | C_27_H_42_O_4_ | 13.52 | 453.2971795 | M+Na | 441893 |
| 413 | EURYCOMANONE | C_20_H_24_O_9_ | 12.66 | 373.1272894 | M+H-2H2O | 13936691 |
| 414 | threo-1-C-Syringylglycerol | C_11_H_16_O_6_ | 6.46 | 227.090997 | M+H-H2O | 75492726 |
| 415 | Isorhamnetin 3-glucuronide | C_22_H_20_O_13_ | 7.99 | 491.0825603 | M-H | 5491630 |
| 416 | meso-dihydroguaiaretic acid | C_20_H_26_O_4_ | 12.51 | 353.169173 | M+Na | 476856 |
| 417 | Irilone | C_16_H_10_O_6_ | 8.94 | 343.0453253 | M+FA-H | 5281779 |
| 418 | Hispidulin | C_16_H_12_O_6_ | 10.48 | 301.070169 | M+H, M+Na | 5281628 |
| 419 | trans-2-Tridecene-1,13-dioic acid | C_13_H_22_O_4_ | 11.98 | 241.1440437 | M-H | 23091819 |
| 420 | Pierisformoside B | C_26_H_42_O_8_ | 11.09 | 505.2763208 | M+Na | 155978780 |
| 421 | naringenin chalcone | C_15_H_12_O_5_ | 10.34 | 273.0751479 | M+H | 5280960 |
| 422 | Cearoin | C_14_H_12_O_4_ | 11.04 | 225.0555203 | M-H2O-H | 3938139 |
| 423 | p-Hydroxy-5,6-dehydrokawain | C_14_H_12_O_4_ | 10.29 | 225.0554497 | M-H2O-H | 10243535 |
| 424 | Nardosinone | C_15_H_22_O_3_ | 10.25 | 273.1457544 | M+Na | 168136 |
| 425 | Methylmalonic acid | C_4_H_6_O_4_ | 5.22 | 278.0877498 | 2M+ACN+H | 487 |
| 426 | Inulicin | C_17_H_24_O_5_ | 9.51 | 350.1962604 | M+ACN+H | 75528891 |
| 427 | 20(S),24(R)-Ocotillol | C_30_H_52_O_5_ | 14.32 | 515.3702324 | M+Na | 15886258 |
| 428 | Robinetin | C_15_H_10_O_7_ | 8.93 | 303.0492067 | M+H | - |
| 429 | Citrate | C_6_H_8_O_7_ | 3.70 | 210.061149 | M+NH4 | 31348 |
| 430 | Ehretioside B | C_14_H_17_NO_7_ | 5.07 | 312.1069857 | M+H | 10425556 |
| 431 | Dracoflavan B_1_ | C_33_H_30_O_7_ | 12.09 | 503.1876257 | M+H-2H2O | 177391 |
| 432 | toralactone | C_15_H_12_O_5_ | 12.50 | 271.0608606 | M-H | 5321980 |
| 433 | Flavin mononucleotide | C_17_H_19_N_4_O_6_ | 13.13 | 393.1655576 | M+NH4 | 643976 |
| 434 | Eupatilin | C_18_H_16_O_7_ | 11.91 | 343.0815306 | M-H | 5273755 |
| 435 | Prunasin | C_14_H_17_NO_6_ | 4.64 | 337.138708 | M+ACN+H | 119033 |
| 436 | Luteolin 7-glucuronide | C_21_H_18_O_12_ | 9.51 | 461.0720815 | M-H, M+Na-2H | 5282153 |
| 437 | Daphnetin | C_9_H_6_O_4_ | 7.56 | 177.0185015 | M-H | 5280569 |
| 438 | Ferulamide | C_10_H_11_NO_3_ | 6.19 | 238.0715898 | M+FA-H | 6433734 |
| 439 | Jasmonic acid | C_12_H_18_O_3_ | 8.54 | 252.1591487 | M+ACN+H | 5281166 |
| 440 | 6''-O-Acetylglycitin | C_24_H_24_O_11_ | 8.77 | 533.1299643 | M+FA-H | 10228095 |
| 441 | Preisocalamendiol | C_15_H_24_O | 10.54 | 203.1792609 | M+H-H2O | 12305706 |
| 442 | Huperzine A | C_15_H_18_N_2_O | 9.10 | 241.1338597 | M-H | 854026 |
| 443 | Frangulin B | C_20_H_18_O_9_ | 9.70 | 403.1014654 | M+H | 442744 |
| 444 | Isovanillic acid | C_8_H_8_O_4_ | 4.39 | 213.039849 | M+FA-H | 12575 |
| 445 | Cryptochlorogenic acid | C_16_H_18_O_9_ | 7.34 | 335.0769332 | M-H2O-H | 9798666 |
| 446 | Virginiaebutanolide A | C_12_H_22_O_4_ | 11.23 | 229.1438657 | M-H | 40561590 |
| 447 | Planchol E | C_14_H_12_O_7_ | 11.29 | 273.0400761 | M-H2O-H | 53350155 |
| 448 | Isoanhydroicaritin | C_21_H_20_O_6_ | 9.96 | 369.1313469 | M+H | 5322079 |
| 449 | Microminutin | C_15_H_12_O_5_ | 8.91 | 317.0662316 | M+FA-H | 5319827 |
| 450 | Traumatic Acid | C_12_H_20_O_4_ | 11.86 | 227.1283315 | M-H | 5283028 |
| 451 | Bergaptol glucoside | C_17_H_16_O_9_ | 10.54 | 329.0648587 | M+H-2H2O | - |
| 452 | Giffonin R | C_19_H_16_O_3_ | 10.74 | 257.0956275 | M+H-2H2O | 134715258 |
| 453 | [(3,8,12-trihydroxy-24-oxocholan-24-yl)amino]acetate | C_26_H_42_NO_6_^-^ | 10.54 | 509.2716424 | M+2Na-H | 11834768 |
| 454 | Peucedanol | C_14_H_16_O_5_ | 9.44 | 247.0938444 | M+H-H2O | 15296614 |
| 455 | De-O-methylprosolanapyrone I | C_17_H_22_O_3_ | 9.12 | 319.1281989 | M+2Na-H | 102077066 |
| 456 | (E)-Cinnamic acid | C_9_H_8_O_2_ | 8.51 | 147.0441142 | M-H | 444539 |
| 457 | Endocrocin | C_16_H_10_O_7_ | 11.48 | 313.0349092 | M-H | 160483 |
| 458 | Isofraxidin | C_11_H_10_O_5_ | 8.79 | 264.0860739 | M+ACN+H | 5318565 |
| 459 | Purpurogallin | C_11_H_8_O_5_ | 6.09 | 221.0434543 | M+H | 135403797 |
| 460 | Ursolic acid | C_30_H_48_O_3_ | 14.74 | 457.3665571 | M+H | - |
| 461 | Alpha-Asarone | C_12_H_16_O_3_ | 11.17 | 253.1077073 | M+FA-H | 636822 |
| 462 | Lucidone | C_15_H_12_O_4_ | 9.51 | 301.0713671 | M+FA-H | 11253859 |
| 463 | Cordycepin | C_10_H_13_N_5_O_3_ | 4.20 | 252.1085735 | M+H | 6303 |
| 464 | Dihydrophaseic acid | C_15_H_22_O_5_ | 7.69 | 300.1798748 | M+NH4 | 11988272 |
| 465 | 3,5-DIHYDROXY-4-METHOXYBENZOIC ACID | C_8_H_8_O_5_ | 8.06 | 183.0290153 | M-H | - |
| 466 | Usnic acid | C_18_H_16_O_7_ | 14.26 | 343.0816788 | M-H | 442614 |
| 467 | Taxifoliol | C_15_H_12_O_7_ | 5.23 | 303.0506271 | M-H | 439533 |
| 468 | Laetisaric acid | C_18_H_32_O_3_ | 14.82 | 261.2206176 | M+H-2H2O | 5281117 |
| 469 | Hydrourushiol | C_21_H_36_O_2_ | 13.13 | 365.2689119 | M+FA-H | 68118 |
| 470 | kaempferol | C_15_H_10_O_6_ | 9.66 | 287.0544213 | M+H | 5280863 |
| 471 | Danshenol C | C_21_H_20_O_4_ | 11.52 | 301.1216489 | M+H-2H2O, M+CH3OH+H, M+H-H2O | - |
| 472 | 1,4-Dihydroxy-2-carbomethoxy-3-prenylnaphthalene-1-O-beta-glucopyranoside | C_23_H_28_O_9_ | 10.77 | 481.2050333 | M+CH3OH+H | 138857786 |
| 473 | baicalein | C_15_H_10_O_5_ | 8.80 | 271.0595687 | M+H | 5281605 |
| 474 | Kaempferol 3-glucorhamnoside | C_27_H_30_O_15_ | 9.51 | 593.1484539 | M-H | 127256226 |
| 475 | 2-O-Methylanigorufone | C_20_H_14_O_2_ | 11.52 | 269.0956223 | M+H-H2O | - |
| 476 | Kirenol | C_20_H_34_O_4_ | 13.65 | 339.2499407 | M+H, M+2Na-H | 15736732 |
| 477 | Protocetraric acid | C_18_H_14_O_9_ | 10.67 | 357.0594621 | M+H-H2O | 5489486 |
| 478 | 3-O-Ethyl-L-ascorbic acid | C_8_H_12_O_6_ | 5.89 | 185.044761 | M-H2O-H | 150736 |
| 479 | 2-Methoxynaphthoquinone | C_11_H_8_O_3_ | 8.95 | 233.0451658 | M+FA-H | 16871 |
| 480 | Gardoside | C_16_H_22_O_10_ | 10.03 | 339.1066991 | M+H-2H2O | 46173850 |
| 481 | Dihydrosesamin | C_20_H_20_O_6_ | 9.30 | 355.1182984 | M-H | 10871980 |
| 482 | 1_1_S,12-Dihydroxyspirovetiv-1(10)-en-2-one | C_15_H_24_O_3_ | 12.63 | 251.1648879 | M-H | - |
| 483 | Esculetin | C_9_H_6_O_4_ | 7.55 | 179.0337236 | M+H, M+Na | 5281416 |
| 484 | Citropten | C_11_H_10_O_4_ | 8.25 | 251.0556509 | M-H2O-H, M+FA-H | 2775 |
| 485 | Massarilactone B | C_11_H_14_O_5_ | 7.70 | 207.0656457 | M-H2O-H | 10105097 |
| 486 | Kumatakenin | C_17_H_14_O_6_ | 7.89 | 337.0698206 | M+Na | 5318869 |
| 487 | cinnamaldehyde | C_9_H_8_O | 11.00 | 133.0647974 | M+H | 637511 |
| 488 | 3-O-Acetylpinobanksin | C_17_H_14_O_6_ | 7.49 | 337.0697456 | M+Na | 148556 |
| 489 | gamma-Linolenic acid | C_18_H_30_O_2_ | 13.81 | 323.2221263 | M+FA-H | 5280933 |
| 490 | Desmethylbellidifolin | C_13_H_8_O_6_ | 10.16 | 259.0244757 | M-H | 5281626 |
| 491 | Cedrin | C_16_H_14_O_8_ | 9.90 | 317.0648106 | M+H-H2O | 21721881 |
| 492 | Ethyl alpha-D-apiofuranoside | C_7_H_14_O_5_ | 1.37 | 161.08072 | M+H-H2O | - |
| 493 | emodin | C_15_H_10_O_5_ | 13.42 | 269.0452968 | M-H | 3220 |
| 494 | Juncuenin D | C_18_H_18_O_3_ | 10.46 | 327.0967655 | M+2Na-H | 44178856 |
| 495 | Isorhamnetin | C_16_H_12_O_7_ | 11.54 | 315.050669 | M-H | 5281654 |
| 496 | Magnolioside | C_16_H_18_O_9_ | 8.93 | 319.0805195 | M+H-2H2O | 3084335 |
| 497 | Asperuloside | C_18_H_22_O_11_ | 7.10 | 413.1085316 | M-H | 84298 |
| 498 | 2-Prenylhydroquinone-1-glucoside | C_17_H_24_O_7_ | 8.60 | 361.12661 | M+Na-2H | - |
| 499 | 5'-DEOXYADENOSINE | C_10_H_13_N_5_O_3_ | 1.64 | 272.0772783 | M+Na-2H | 439182 |
| 500 | 8-Methoxybonducellin | C_18_H_16_O_5_ | 8.90 | 357.097652 | M+FA-H | 73353608 |
| 501 | Neposide | C_19_H_22_O_8_ | 8.33 | 359.1135459 | M-H2O-H | 12313291 |
| 502 | Bergapten | C_12_H_8_O_4_ | 10.44 | 261.0401663 | M+FA-H | 2355 |
| 503 | 3-Chloro-1-(4-octylphenyl)-propanone | C_17_H_25_ClO | 13.97 | 263.1570544 | M+H-H2O | 70700719 |
| 504 | Pterosin C | C_14_H_18_O_3_ | 9.36 | 279.123363 | M+FA-H | 186209 |
| 505 | Phyllanthurinolactone | C_14_H_18_O_8_ | 8.07 | 353.0645329 | M+K | 10957981 |
| 506 | 5-Methyl-7-methoxyisoflavone | C_17_H_14_O_3_ | 11.22 | 231.080128 | M+H-2H2O | 2734290 |
| 507 | quercetin | C_15_H_10_O_7_ | 8.69 | 303.0493343 | M+H | 5280343 |
| 508 | Scopolin | C_16_H_18_O_9_ | 7.34 | 377.0834591 | M+Na | 439514 |
| 509 | Demethoxycurcumin | C_20_H_18_O_5_ | 12.09 | 339.1220051 | M+H, M+Na | 5469424 |
| 510 | Ochratoxin B | C_20_H_19_NO_6_ | 6.93 | 370.127265 | M+H | 20966 |
| 511 | alpha-Isowighteone | C_20_H_18_O_5_ | 12.08 | 303.1009337 | M+H-2H2O | 91885205 |
| 512 | Viscidulin I | C_15_H_10_O_7_ | 9.04 | 301.0346011 | M-H | 5320471 |
| 513 | 7-Methoxycoumarin | C_10_H_8_O_3_ | 10.11 | 221.0449983 | M+FA-H | 10748 |
| 514 | Xanthopterin | C_6_H_5_N_5_O_2_ | 5.55 | 403.0881518 | 2M+FA-H | - |
| 515 | Altamisic acid | C_15_H_20_O_5_ | 10.35 | 261.1128975 | M-H2O-H | 15143694 |
| 516 | Deacetylorientalide | C_19_H_22_O_7_ | 10.81 | 401.0987584 | M+K | 23815409 |
| 517 | Picrotoxinin | C_15_H_16_O_6_ | 9.54 | 273.0766417 | M-H2O-H | 442292 |
| 518 | artemisinin | C_15_H_22_O_5_ | 8.93 | 300.1798502 | M+NH4 | 68827 |
| 519 | Paeoniflorigenone | C_17_H_18_O_6_ | 9.90 | 301.1063847 | M+H-H2O | - |
| 520 | Altholactone | C_13_H_12_O_4_ | 8.94 | 277.0713375 | M+FA-H | 442513 |
| 521 | Hexylitaconic acid | C_11_H_18_O_4_ | 11.67 | 213.1126171 | M-H | 11447214 |
| 522 | Erythbidin A | C_20_H_20_O_4_ | 11.69 | 323.1284103 | M-H | 15391906 |
| 523 | Eupalinilide B | C_20_H_24_O_6_ | 8.74 | 361.1612675 | M+H | 11245337 |
| 524 | Bisdemethoxycurcumin | C_19_H_16_O_4_ | 12.06 | 309.1114048 | M+H, M+Na | 5315472 |
| 525 | Eleutherazine B | C_22_H_36_N_4_O_6_ | 7.22 | 453.2697844 | M+H, M+Na | 20839739 |
| 526 | Clausenin | C_14_H_12_O_5_ | 9.98 | 241.0501985 | M-H2O-H | 5315948 |
| 527 | Excavatin M | C_19_H_20_O_7_ | 7.81 | 359.1132749 | M-H | 15871351 |
| 528 | 7-Methoxy-1-naphthaleneacetic acid | C_13_H_12_O_3_ | 8.29 | 197.0601329 | M-H2O-H | 6862 |
| 529 | Ethyl brevifolincarboxylate | C_15_H_12_O_8_ | 12.36 | 338.0870948 | M+NH4 | - |
| 530 | Retusin | C_19_H_18_O_7_ | 9.29 | 341.1010075 | M+H-H2O, M+H | 5352005 |
| 531 | 1-(3,4-Dihydroxyphenyl)-7-(4-hydroxyphenyl)-4-hept | C_19_H_20_O_4_ | 11.18 | 295.1321871 | M+H-H2O | 11570978 |
| 532 | Carpinontriol B | C_19_H_20_O_6_ | 9.63 | 389.1237799 | M+FA-H | 85374891 |
| 533 | Eriodictyol | C_15_H_12_O_6_ | 5.19 | 289.0700333 | M+H | 440735 |
| 534 | Pinocembrin diacetate | C_19_H_16_O_6_ | 9.42 | 385.092484 | M+FA-H | 6546286 |
| 535 | 5-O-Cinnamoylquinic acid | C_16_H_18_O_7_ | 8.97 | 345.0960764 | M+Na | 162642276 |
| 536 | Curdione | C_15_H_24_O_2_ | 4.57 | 259.1646793 | M+Na | 6441391 |
| 537 | Verbasoside | C_20_H_30_O_12_ | 7.61 | 485.1619805 | M+Na | 11754080 |
| 538 | 2-Methoxystypandrone | C_14_H_12_O_5_ | 8.69 | 259.0608847 | M-H | 158739 |
| 539 | Demethylwedelolactone | C_15_H_8_O_7_ | 9.04 | 299.0192017 | M-H | 5489605 |
| 540 | Nortanshinone | C_17_H_12_O_4_ | 10.92 | 325.0713424 | M+FA-H | 10062187 |
| 541 | Tutin | C_15_H_18_O_6_ | 7.74 | 259.0960122 | M+H-2H2O | 75729 |
| 542 | Armillarisin A | C_12_H_10_O_5_ | 6.70 | 279.050605 | M+FA-H | 5320192 |
| 543 | Citrusinol | C_20_H_16_O_6_ | 10.36 | 317.0801047 | M+H-2H2O | 44259051 |
| 544 | Avicularin | C_20_H_18_O_11_ | 9.25 | 433.0771161 | M-H | 5490064 |
| 545 | Verminoside | C_24_H_28_O_13_ | 8.26 | 569.1252454 | M+2Na-H | 12000883 |
| 546 | Praeruptorin A | C_21_H_22_O_7_ | 9.01 | 409.1248149 | M+Na | 38347607 |
| 547 | Hamaudol | C_15_H_16_O_5_ | 11.48 | 241.0855557 | M+H-2H2O | 164722 |
| 548 | Gardenine | C_11_H_13_NO_4_ | 9.56 | 258.053229 | M+Cl | 197414 |
| 549 | 1,2-Didehydrocryptotanshinone | C_19_H_18_O_3_ | 11.38 | 339.1231765 | M+FA-H | - |
